# Myonuclear Domain‐Associated and Central Nucleation‐Dependent Spatial Restriction of Dystrophin Protein Expression

**DOI:** 10.1002/jcsm.70284

**Published:** 2026-04-07

**Authors:** Katarzyna Chwalenia, Vivi‐Yun Feng, Nicole Hemmer, John C. W. Hildyard, Liberty E. Roskrow, Richard J. Piercy, Eric T. Wang, Annemieke Aartsma‐Rus, Maaike van Putten, Matthew J. A. Wood, Thomas C. Roberts

**Affiliations:** ^1^ Department of Paediatrics University of Oxford Oxford UK; ^2^ Institute of Developmental and Regenerative Medicine University of Oxford Oxford UK; ^3^ Freie Universität Berlin Berlin Germany; ^4^ Eberhard‐Karls‐Universität Tübingen, Geschwister‐Scholl‐Platz Tübingen Germany; ^5^ Comparative Neuromuscular Diseases Laboratory, Department of Clinical Science and Services Royal Veterinary College London UK; ^6^ Department of Molecular Genetics & Microbiology, Center for NeuroGenetics, Genetics Institute University of Florida Gainesville Florida USA; ^7^ Myology Institute University of Florida Gainesville Florida USA; ^8^ Department of Human Genetics Leiden University Medical Center Leiden the Netherlands; ^9^ MDUK Oxford Neuromuscular Centre Oxford UK

**Keywords:** central nucleation, DMD, dystrophin, myonuclear domain, X‐chromosome inactivation

## Abstract

**Background:**

The restoration of uniformly distributed dystrophin protein expression is an important consideration for the development of advanced therapeutics for Duchenne muscular dystrophy (DMD).

**Methods:**

We have generated a novel genetic mouse model (*mdx52‐Xist* ^Δhs^) that expresses variable and nonuniformly distributed dystrophin protein from birth as a consequence of skewed X‐chromosome inactivation. *mdx52‐Xist* ^Δhs^ myofibers are heterokaryons containing a mixture of myonuclei expressing either wild‐type or mutant dystrophin alleles in a mutually exclusive manner, resulting in dystrophin protein being spatially restricted to corresponding dystrophin‐expressing myonuclear domains. This phenotype models the situation in female dystrophinopathy and dystrophic muscle in which dystrophin has been incompletely restored by partially effective experimental therapeutics. Dystrophin distribution was assessed in *mdx52‐Xist* ^Δhs^ muscle sections and isolated single myofibers by immunostaining and RNA‐FISH analysis.

**Results:**

Total dystrophin expression increased by ~2.8‐fold (*p* < 0.010) in aged (60‐week‐old) *mdx52‐Xist* ^Δhs^ mice relative to 6‐week‐old adults, suggestive of an aging‐associated accumulation of dystrophin‐expressing myonuclei through positive selection, although this was insufficient to resolve sarcolemmal dystrophin patchiness. Nonuniformly distributed dystrophin conferred partial protection against pathology‐related muscle turnover in an expression‐level‐dependent manner in both adult and aged *mdx52‐Xist* ^Δhs^ mice compared to mice expressing no dystrophin. Isolated *mdx52‐Xist* ^Δhs^ myofibers exhibited patchy ‘zebra‐like’ banding of dystrophin sarcolemmal coverage that colocalized with *β*‐dystroglycan but not neuronal nitric oxide synthase, which was uniformly distributed.

Systematic classification of isolated *mdx52‐Xist* ^Δhs^ myofibers revealed unexpected and profound differences associated with central nucleation, with dystrophin found to be absent in centrally nucleated myofibers and myofiber segments. Muscle injury alone was insufficient to recapitulate this phenomenon, suggesting that it is a feature of the dystrophic environment. *Dmd* mRNA was found to be present throughout centrally nucleated segments, and proteins such as titin and F‐actin were uniformly distributed, suggesting that dystrophin is specifically repressed at the protein level in these regions. The microtubule network was moderately disrupted in *mdx52‐Xist* ^Δhs^ ‘zebra‐banded’ fibers, but this effect was not different between dystrophin‐positive and dystrophin‐negative myofiber subdomains. By contrast, centrally nucleated *mdx52‐Xist* ^Δhs^ myofibers exhibited severe microtubule network disruption.

**Conclusions:**

These findings reveal new insights into the importance of dystrophin spatial localization and identify a previously unappreciated barrier to effective therapeutic dystrophin restoration, particularly within regenerated or centrally nucleated myofibers.

## 1 Introduction

DMD is a monogenic muscle wasting disorder caused by pathogenic variants (frequently whole exon deletions) in the *DMD* gene, which encodes the dystrophin protein. Dystrophin forms a mechanical link between the cytoskeleton (i.e., filamentous actin and microtubules) and the extracellular matrix via interactions with components of the dystrophin‐associated protein complex (DAPC), which consists of both structural and signalling proteins. The absence of dystrophin protein at the sarcolemma, and subsequent disruption of DAPC assembly, sensitizes muscle to contraction‐induced damage [1]. In DMD patients, this leads to repeated muscle turnover, persistent inflammation and the progressive replacement of myocytes with fatty and fibrotic tissue.

While still rare, the relatively high prevalence of the disorder (~1 in 5 000 males) and the severity of the disease have made DMD a priority candidate for experimental therapeutics. Indeed, there are now four antisense oligonucleotide (ASO) drugs and one microdystrophin gene therapy that have received (accelerated) marketing authorization from the US FDA [2]. Furthermore, a multitude of other approaches are under investigation, including CRISPR‐Cas9‐mediated gene editing and upregulation of utrophin (a dystrophin paralogue) [2, 3, 4, 5, 6, 7]. Despite this progress, the clinical challenge of effectively treating DMD remains incompletely met, in part due to a combination of poor drug delivery, incomplete functionality of the restored internally deleted quasi‐dystrophin protein and failure to rescue dystrophin in all fibers. We have observed that the pattern of dystrophin expression restored following treatment is dependent on the modality. Specifically, we observed a uniform sarcolemmal pattern of dystrophin expression following treatment of the *mdx* mouse model of DMD with peptide‐phosphorodiamidate morpholino oligonucleotides (PPMOs) designed to induce exon skipping of *Dmd* exon 23 (containing a premature termination codon) [8, 9] and a patchy pattern of dystrophin expression following CRISPR‐Cas9‐mediated excision of the same exon in the severely affected dystrophin/utrophin double knock‐out (dKO) mouse [10]. Similar results were also reported by Morin et al. [11] Importantly, analysis of human biopsies has shown that incomplete sarcolemmal dystrophin coverage correlates with pathological severity in Becker muscular dystrophy and intermediate muscular dystrophy patients (i.e., those with clinical phenotypes that are between those of BMD and DMD) [12]. Spatial restriction of dystrophin can be attributed to its limited capacity to diffuse throughout the sarcolemma, such that it becomes localized in the vicinity of its corresponding myonucleus of origin, consistent with the myonuclear domain hypothesis [13].

We have previously modelled the effects of patchy sarcolemmal dystrophin expression using skewed X‐chromosome inactivation (XCI) in a murine system [9, 14, 15]. To further investigate this patchy dystrophin phenomenon, we have developed a novel mouse model that exhibits preferential XCI of the healthy X‐chromosome, while the mutated X‐chromosome carries a patient‐relevant whole exon deletion of *Dmd* exon 52 [16]. Using this novel system, we show that the female *mdx52‐Xist* ^Δhs^ mice exhibit variable levels of dystrophin expression with a characteristic patchy pattern of dystrophin coverage at the sarcolemma. Comparison of *mdx52‐Xist* ^Δhs^ mice at adult (6 week) and aged (60 week) time points revealed an overall increase in total dystrophin level, consistent with the accumulation of dystrophin‐positive myofibers with time. However, this increase in dystrophin expression was insufficient to resolve sarcolemmal dystrophin patchiness, suggesting that these fibers are incompletely protected from the cycles of myonecrosis and compensatory regeneration that are characteristic pathological features of DMD. However, myofiber central nucleation was inversely correlated with total dystrophin protein levels, suggesting that patchy dystrophin expression does offer myofibers a degree of protection. Interestingly, analysis of *mdx52‐Xist* ^Δhs^ isolated single myofibers revealed that centrally nucleated myofibers and myofiber segments were almost completely devoid of dystrophin or DAPC expression. This unexpected finding was attributed to local and specific inhibition of dystrophin expression at the protein level. This study has important implications for therapeutic efforts to restore dystrophin protein expression in Duchenne patients.

## 2 Methods

### 2.1 Animal Studies

All experimental procedures were approved by the UK home office, under the project licence number PP6777529 (Oxford) or PPL 70/7777 (RVC, approved by the Royal Veterinary College Animal Welfare and Ethical Review Board), in accordance with the Animals (Scientific Procedures) Act 1986. Animals were housed in individually ventilated cages with a 12:12 h light:dark cycle, with food and water provided *ad libitum*. Details of animal strains and BaCl_2_ myoinjury are provided in Supporting Information.

### 2.2 Protein Quantification

Dystrophin protein quantification was performed on tibialis anterior (TA) lysates using a standard western blotting protocol (Supporting Information). Briefly, protein was extracted from 200 TA sections (8 μm thickness), separated by polyacrylamide gel electrophoresis and immunoblotted using antibodies as described in Table S1 and Table S2.

### 2.3 Single Fiber Isolation

Extensor digitorum longus (EDL) single myofiber isolation was performed as described previously [17]. Briefly, EDL muscles were dissected tendon‐to‐tendon and incubated in 0.2% collagenase II (Worthington, NJ, USA) diluted in filter‐sterilized DMEM (Thermo Fisher Scientific, prewarmed at 37°C) for 45–52 min at 37°C. Digestion was stopped by transferring the muscle into a 3.5‐cm cell culture dish, containing FluoroBrite DMEM media (Thermo Fisher Scientific) supplemented with 1% Antibiotic‐Antimycotic (PSA: Penicillin, Streptomycin and Amphotericin B; Thermo Fisher Scientific) prewarmed at 37°C. Single myofibers were released from the muscle by gentle flushing using a 200 μL pipette under a stereomicroscope. Freshly isolated myofibers were transferred into a spot plate containing 4% ultrapure paraformaldehyde solution (PFA, Electron Microscopy Sciences, PA, USA) for fixation for 10 min at room temperature. Fixed myofibers were washed twice with ultrapure PBS for 5 min at room temperature. Immunofluorescence and/or hybridization chain reaction‐based RNA in situ hybridisation (HCR‐RNA‐FISH) were performed immediately after fixation and PBS washes.

### 2.4 Immunofluorescence in Tissue Sections

Fresh frozen TA muscles were mounted onto corks with Tissue‐TEK optimal cutting temperature (OCT) Compound (Sakura, Japan) and cryosectioned (8 μm) in transverse and longitudinal orientations. Samples were stored at −80°C prior to analysis. On the day of staining, slides were air‐dried and soaked in phosphate‐buffered saline (PBS, Thermo Fisher Scientific) for 10 min at room temperature. Sections were blocked in blocking buffer composed of PBS supplemented with 20% foetal calf serum (FCS, Thermo Fisher Scientific) and 20% normal goat serum (NGS, MP Biomedicals, CA, USA) for 2 h at room temperature. Subsequently, slides were incubated with primary antibodies (listed in Table S1) in blocking buffer for 2 h at room temperature. After washing three times with PBS, slides were incubated with secondary fluorescent antibodies (Table S2) in PBS or blocking buffer for 1 h at room temperature in darkness. Slides were then washed three times with PBS, incubated with 4′,6‐diamidino‐2‐phenylindole (DAPI) or Hoechst in PBS (1:5 000, Thermo Fisher Scientific), washed with PBS once more and mounted using Dako, Fluorescence Mounting Medium (Agilent Technologies, CA, USA) or SlowFade Diamond Antifade Mountant (Thermo Fisher Scientific).

### 2.5 Immunofluorescence in Isolated Fibers

PFA‐fixed myofibers were permeabilized with 1% Triton‐X100 for 10 min and washed with PBS. Blocking was performed for 30 min in 1% BSA, with RiboLock RNase Inhibitor included if samples were subsequently processed for HCR RNA‐FISH. Myofibers were incubated with primary antibodies (Table S1) for 2 h, washed in PBST and then incubated with secondary fluorescent antibodies (Table S2) for 2 h at room temperature.

For protein‐only detection, fibers were washed, counterstained with DAPI, mounted with antifade medium and imaged or stored at −20°C. For combined protein and RNA‐FISH detection, samples were washed once with PBS and refixed in 4% PFA before proceeding with HCR RNA‐FISH (see below).

### 2.6 RNA‐FISH

RNA‐FISH was performed using the Hybridization Chain Reaction method from Molecular Instruments (Los Angeles, CA, USA) according to the manufacturer's instructions, with some modifications (described in detail in Supporting Information).

### 2.7 Microscopy

Immunofluorescence microscopy of tissue sections was performed using either wide‐field Leica DMIRB Inverted Microscope with MetaMorph imaging software (Molecular Devices, CA, USA) or wide‐field Leica DMi8 fluorescence microscope with LAS X Microscope Science Software Platform (all Leica Microsystems, Wetzlar, Germany). For each protein staining, optimal exposure time was chosen based on negative staining control where samples were incubated with secondary antibodies only to account for background noise and autofluorescence of tissues. All images were processed using Fiji software [18]. Standard image processing for tissue section images included background subtraction (based on rolling ball with radius of 50 pixels) and brightness and contrast adjustment.

Single myofiber imaging was performed with ZEISS LSM 980 confocal microscope with Airyscan2 detector (ZEISS, Oberkochen, Germany). Depending on the application, the following objectives were used: 40× Plan‐Apochromat oil objective (numerical aperture NA = 1.4), 25× Plan‐Apochromat (NA = 0.8) or 20× Plan‐Apochromat (NA = 0.8). The choice of the objective was based on the field of view and detail required in each experiment. Image analysis is described in detail in Supporting Information.

### 2.8 Statistical Analysis

Statistical analyses were performed using GraphPad Prism (v10.2.3) (GraphPad Software Inc., San Diego, California, USA). For comparisons of two groups, a Student's *t*‐test was used. For comparisons of more than two groups, an ordinary one‐way analysis of variance (ANOVA) was performed with Bonferroni's post hoc test for intergroup comparisons.

## 3 Results

### 3.1 Dystrophin Is Expressed in a Within‐Fiber Patchy Manner in Adult *mdx52‐Xist* ^Δhs^ Muscle

To investigate the importance of patchy sarcolemmal dystrophin expression, we generated a novel genetic mouse model called *mdx52‐Xist* ^Δhs^ by crossing male *mdx52* mice with female *Xist* ^Δhs^ mice. The *mdx52* line is a dystrophin‐deficient model that carries a patient‐relevant mutation (whole exon deletion of *Dmd* exon 52) [16, 19]. In the *Xist* ^Δhs^ line, a targeted mutagenesis strategy was used to delete two DNase I hypersensitivity regions upstream of the *Xist* promoter and to replace these with a neomycin resistance cassette [20]. The *Xist* gene encodes a long noncoding RNA that coats its chromosome of origin in *cis* and triggers the formation of facultative chromatin, leading to the transcriptional silencing of genes on the X‐chromosome [21]. The *Xist* ^Δhs^ mutation results in the transcription of *Xist* being increased, leading to premature initiation of XCI of the X‐chromosome during early development [20]. In the context of the *mdx52‐Xist* ^Δhs^ female F1 progeny, this skewed XCI effect results in preferential silencing of the X‐chromosome carrying the healthy *Dmd* allele and is expected to lead to variable levels of dystrophin expressed in a patchy manner (Figure S1) [9, 14, 15]. The *mdx52‐Xist* ^Δhs^ mouse is therefore a model of (i) female dystrophinopathy (previously known as manifesting carriers) [22, 23], and (ii) the situation in dystrophic muscle following partial CRISPR‐Cas9 correction [9, 10].

**FIGURE 1:**
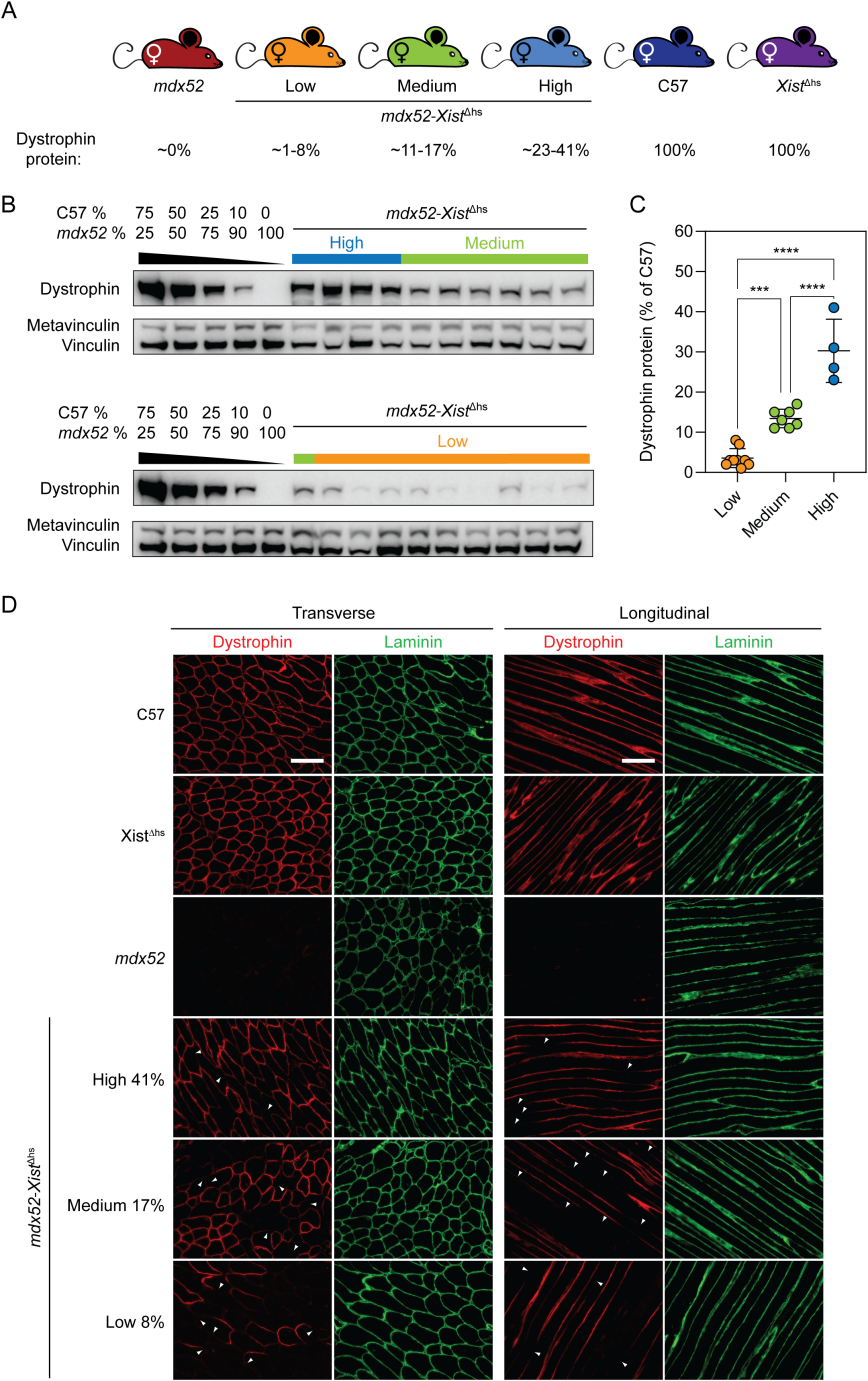
Dystrophin expression is patchy in *mdx52‐Xist* ^∆hs^ muscle. (A) *mdx52‐Xist* ^∆hs^ animals were assigned to high (*n* = 4), medium (*n* = 7) and low (*n* = 9) dystrophin‐expressing groups post‐mortem based on protein quantification in 6‐week‐old tibialis anterior (TA) muscles as determined by (B) Western blot analysis. Wild‐type C57and *Xist* ^Δhs^ mice were used as 100% dystrophin‐expressing controls, and *mdx52* mice used as ~0% dystrophin‐expressing controls. Vinculin was used as a loading control. (C) Dystrophin was quantified by comparison to standard curves containing defined mixtures of C57 and *mdx52* TA lysates. (D) Representative immunofluorescence staining of dystrophin and laminin in transverse and longitudinal TA muscle sections of 6‐week‐old C57 wild‐type, *Xist* ^∆hs^, *mdx52* and *mdx52‐Xist* ^∆hs^ animals from high, medium and low dystrophin‐expressing groups. Within‐fiber, patchy dystrophin expression resulting from skewed X‐chromosome inactivation indicated with arrowheads. Scale bars indicate 100 μm, images taken at 20× magnification. The percentage values indicate total dystrophin quantification in the animals from which the sections were derived. Values are mean ± SD Statistical significance was assessed by one‐way ANOVA with Bonferroni post hoc test, *** *p* < 0.001 and ****p < 0.0001.

Analysis of *mdx52‐Xist* ^Δhs^ females (*N* = 20) revealed a range of total dystrophin expression levels in 6‐week‐old tibialis anterior (TA) muscles and mice were retrospectively assigned to high (~23%–41% of WT dystrophin levels, *n* = 4), medium (~11%–17%, *n* = 7) and low (~1%–8%, *n* = 9) dystrophin‐expressing groups post‐mortem, as determined by western blot (Figure 1A–C). The distributions of dystrophin expression were consistent with those reported in similar studies by our groups [9, 14]. Immunofluorescence analysis in the same tissues revealed a within‐myofiber patchy pattern of sarcolemmal dystrophin (Figure 1D). Regions of adjacent dystrophin‐positive and dystrophin‐negative sarcolemma were observed in the *mdx52*‐*Xist* ^Δhs^ muscles at all dystrophin expression levels. By contrast, dystrophin was uniformly distributed in age‐ and sex‐matched wild‐type C57 and *Xist* ^Δhs^ mice and absent in *mdx52* controls (Figure 1D). Patchiness was more easily visualized in longitudinal sections, but incomplete sarcolemmal coverage was also apparent in some myofibers in transverse sections (especially in the low dystrophin *mdx52‐Xist* ^Δhs^ group). These data show that dystrophin mRNA and protein are not free to diffuse freely within syncytial myofibers, consistent with previous reports [9, 10, 11].

Central nucleation of myofibers in TA transverse sections was strongly inversely correlated with dystrophin expression (Spearman's *r* = −0.86, *p* = 0.0023, Figure S2).

### 3.2 Dystrophin, *β*‐Dystroglycan, and *α*‐Dystrobrevin Are Localized in Sarcolemmal Patches in *mdx52‐Xist* ^Δhs^ Isolated Single Myofibers

**FIGURE 2:**
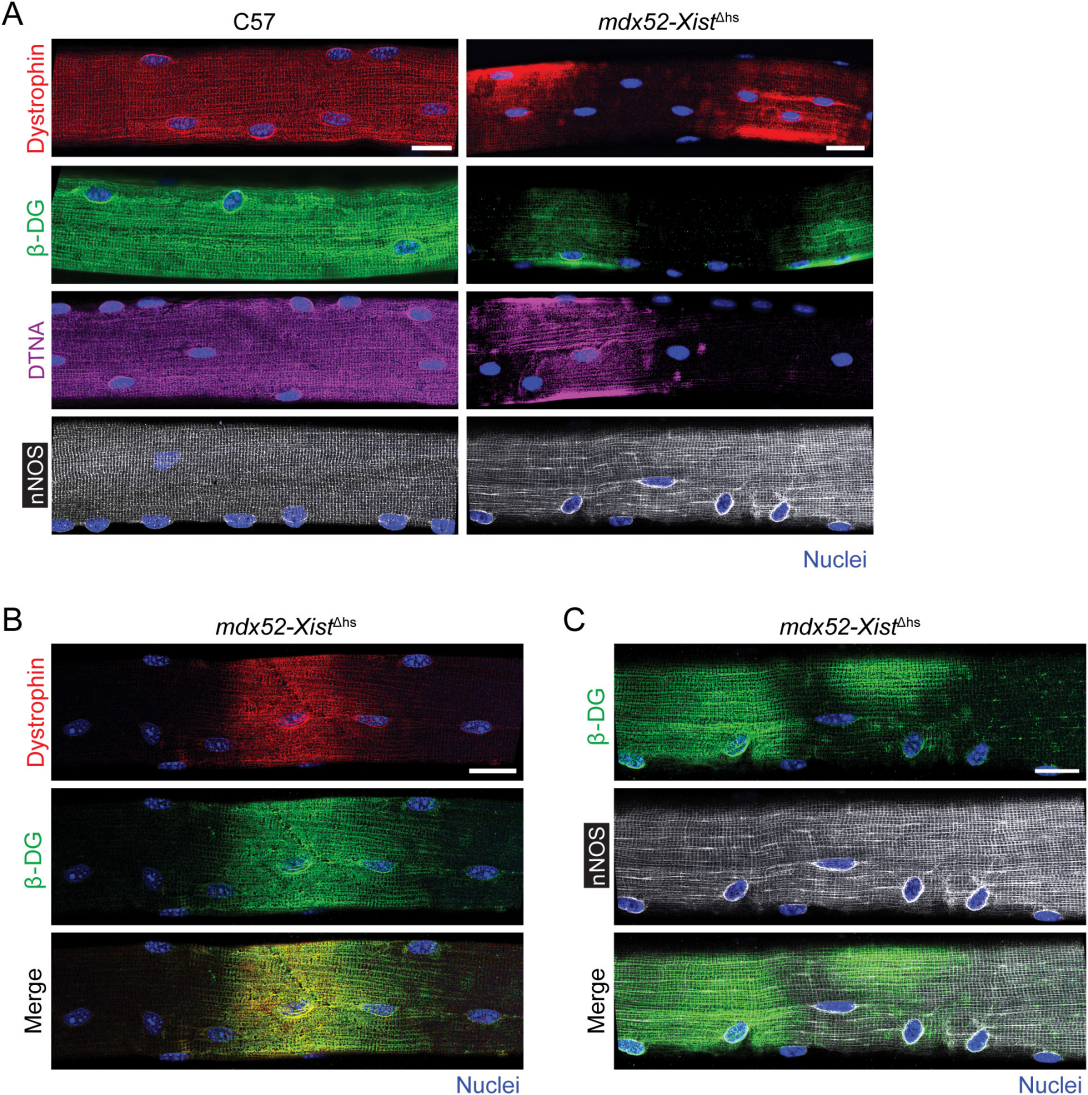
Dystrophin and DAPC protein expression is patchy in *mdx52‐Xist* ^Δhs^ isolated single myofibers. (A) Representative immunofluorescence staining of *β*‐dystroglycan (*β*‐DG), *α*‐dystrobrevin (DTNA) and neuronal nitric oxide synthase (nNOS) in single isolated extensor digitorum longus (EDL) myofibers of adult (6–12 weeks old) C57 (wild‐type) and *mdx52‐Xist* ^Δhs^ mice. Representative costaining images of (B) dystrophin and *β*‐DG, and (C) *β*‐DG and nNOS in the isolated single myofiber of *mdx52‐Xist* ^Δhs^ animals. Scale bars indicates 20 μm, images taken at 25× magnification. Nuclei were stained with DAPI.

Analysis of *mdx52‐Xist* ^Δhs^ isolated single extensor digitorum longus (EDL) myofibers revealed similar patchy sarcolemmal distributions for dystrophin and the DAPC components *β*‐dystroglycan (DAG1) and *α*‐dystrobrevin (DTNA) (Figure 2A). Dual staining showed that dystrophin and *β*‐dystroglycan were colocalized to common regions of the sarcolemma, forming a ‘zebra‐like’ banding pattern of staining (Figure 2B). Conversely, the DAPC protein neuronal nitric oxide synthase (nNOS, NOS1) was uniformly distributed throughout single isolated myofibers derived from both *mdx52‐Xist* ^Δhs^ and wild‐type C57 controls (Figure 2C).

### 3.3 Dystrophin Patchiness Is Maintained in Aged *mdx52‐Xist* ^Δhs^ Muscle

**FIGURE 3:**
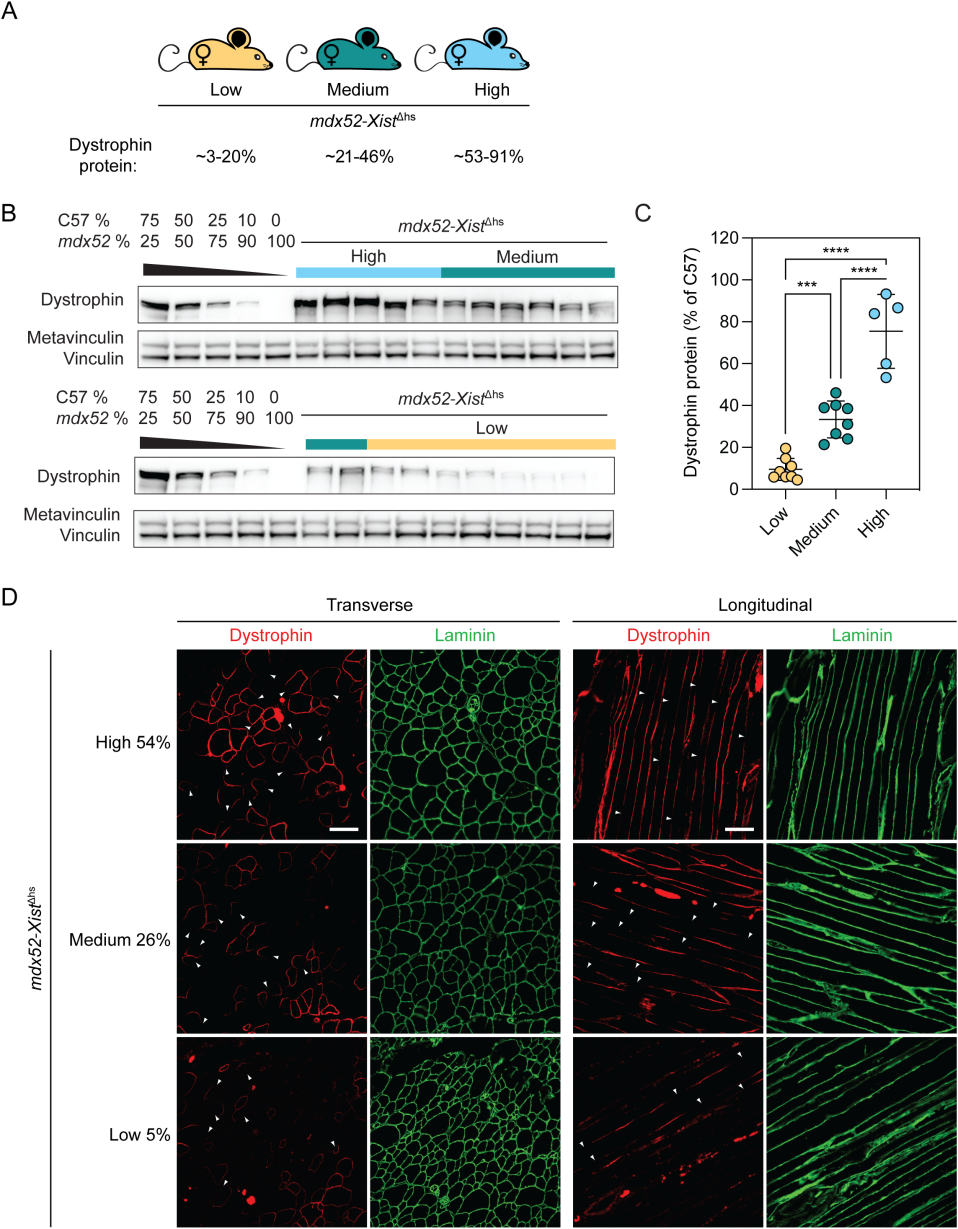
Dystrophin patchiness is maintained in aged *mdx52‐Xist* ^Δhs^ muscle. (A) Aged *mdx52‐Xist* ^∆hs^ animals were assigned to high (*n* = 5), medium (*n* = 8) and low (*n* = 8) dystrophin‐expressing groups post‐mortem based on protein quantification in 60‐week‐old TA muscles as determined by (B) Western blot analysis. (C) Dystrophin was quantified by comparison to standard curves containing defined mixtures of C57 and *mdx52* TA lysates. Vinculin was utilized as a loading control. (D) Representative immunofluorescence staining of dystrophin and laminin in transverse and longitudinal TA muscle sections of 60‐week‐old *mdx52‐Xist* ^∆hs^ animals from high, medium, *β*‐dystroglycan and low dystrophin‐expressing groups. Within‐fiber, patchy dystrophin expression resulting from skewed X‐chromosome inactivation indicated with arrowheads. Scale bars indicate 100 μm, images taken at 20× magnification. The percentage values indicate total dystrophin quantification in the animals from which the sections were derived. Values are mean ± SD Statistical significance was assessed by one‐way ANOVA with Bonferroni post hoc test, ***p < 0.001 and ****p < 0.0001.

It has been proposed that dystrophin protein may accumulate with time following CRISPR‐Cas9‐mediated gene correction as a result of positive selection of corrected, dystrophin‐expressing myofibers [4, 5, 10, 24]. To investigate this dystrophin accumulation phenomenon, we generated a separate cohort of *mdx52‐Xist* ^Δhs^ female F1 mice (*N* = 21) and sacrificed them at 60 weeks of age. Dystrophin expression was determined in TA muscles by western blot in this ‘aged’ cohort and animals retrospectively assigned to high (53%–91% of wild‐type dystrophin, *n* = 5), medium (21%–46%, *n* = 8) and low (3%–20%, *n* = 8) dystrophin expression as described above (Figure 3A–C). The mean dystrophin expression value for all aged *mdx52‐Xist* ^Δhs^ animals was ~2.8‐fold higher than the mean of all 6‐week‐old *mdx52‐Xist* ^Δhs^ animals (*p* < 0.001), consistent with the enrichment of dystrophin‐positive myofibers as a consequence of positive selection. However, the patchy pattern of sarcolemmal dystrophin expression was maintained in aged animals at all dystrophin expression levels (Figure 3C). Myofiber central nucleation was inversely correlated with dystrophin expression (Spearman's *r* = −0.5099, *p* = 0.0257, Figure S3), although the correlation was substantially weaker than that observed for 6‐week‐old animals (Figure S2).

**FIGURE 4:**
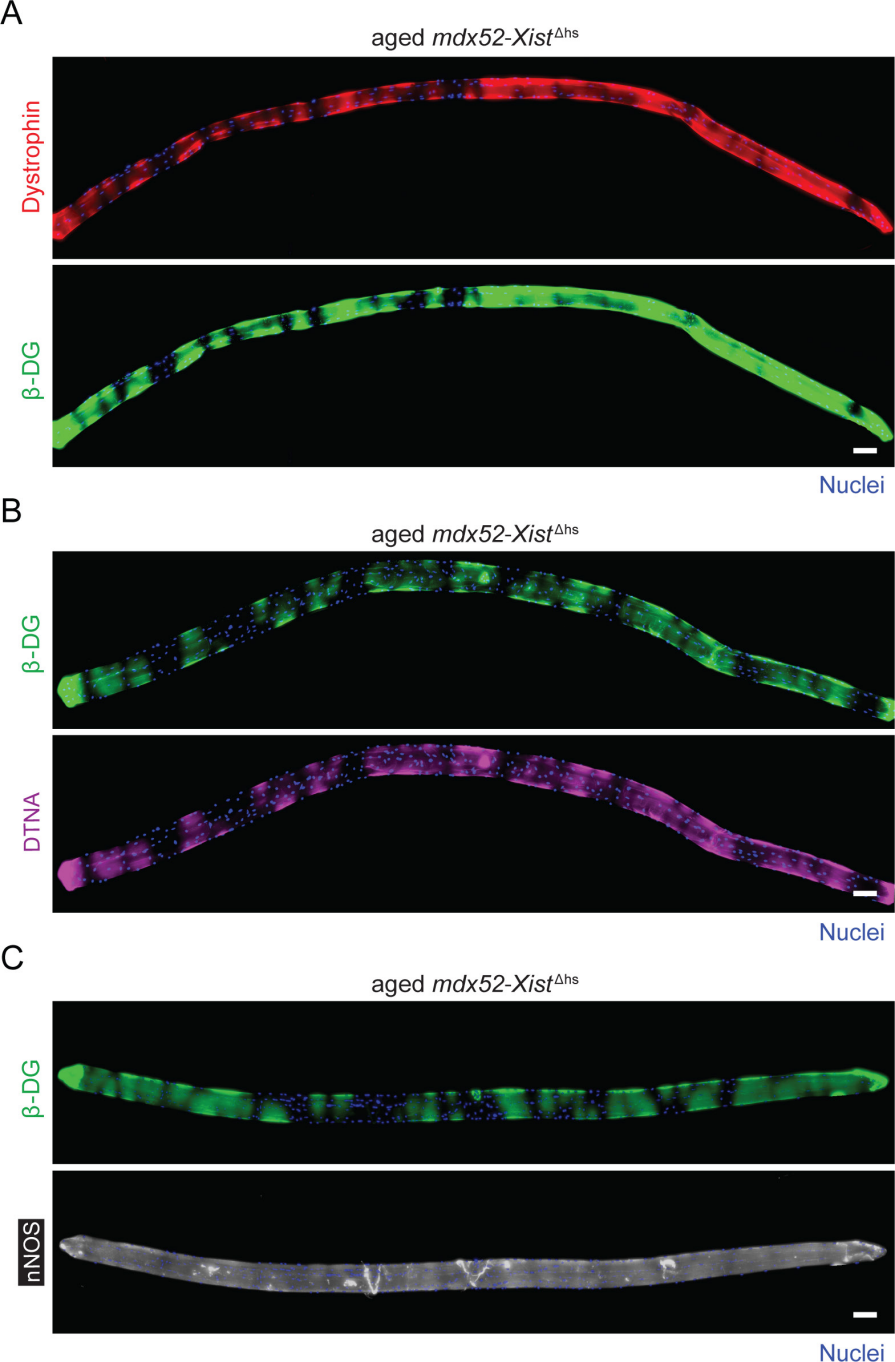
Dystrophin patchiness is maintained in aged *mdx52‐Xist* ^Δhs^ isolated myofibers. Representative immunofluorescence costaining of (A) dystrophin and *β*‐dystroglycan (*β*‐DG), (B) *β*‐DG and *α*‐dystrobrevin (DTNA) and (C) *β*‐DG and nNOS in isolated single 60‐week‐old *mdx52‐Xist* ^Δhs^ EDL myofibers. Tiled images were taken at 10× magnification and stitched together using the LAS X software. Scale bars indicate 100 μm. Nuclei were stained with DAPI.

Analysis of isolated single EDL myofibers from aged *mdx52‐Xist* ^Δhs^ showed that dystrophin, *β*‐dystroglycan (DAG1), and *α*‐dystrobrevin (DTNA) exhibited ‘zebra‐like’ patchy immunostaining patterns, while this effect was much less clear for nNOS (Figure 4), similar to those observations in adult animals (Figure 2).

Notably, utrophin expression was not correlated with dystrophin expression (Figure S4). Serum miRNA biomarkers of muscle regeneration [25] were inversely correlated with dystrophin expression in 6‐week‐old animals, but not in aged animals (Figure S5).

### 3.4 Dystrophin Is Not Expressed in Centrally Nucleated *mdx52‐Xist* ^Δhs^ Segments

**FIGURE 5:**
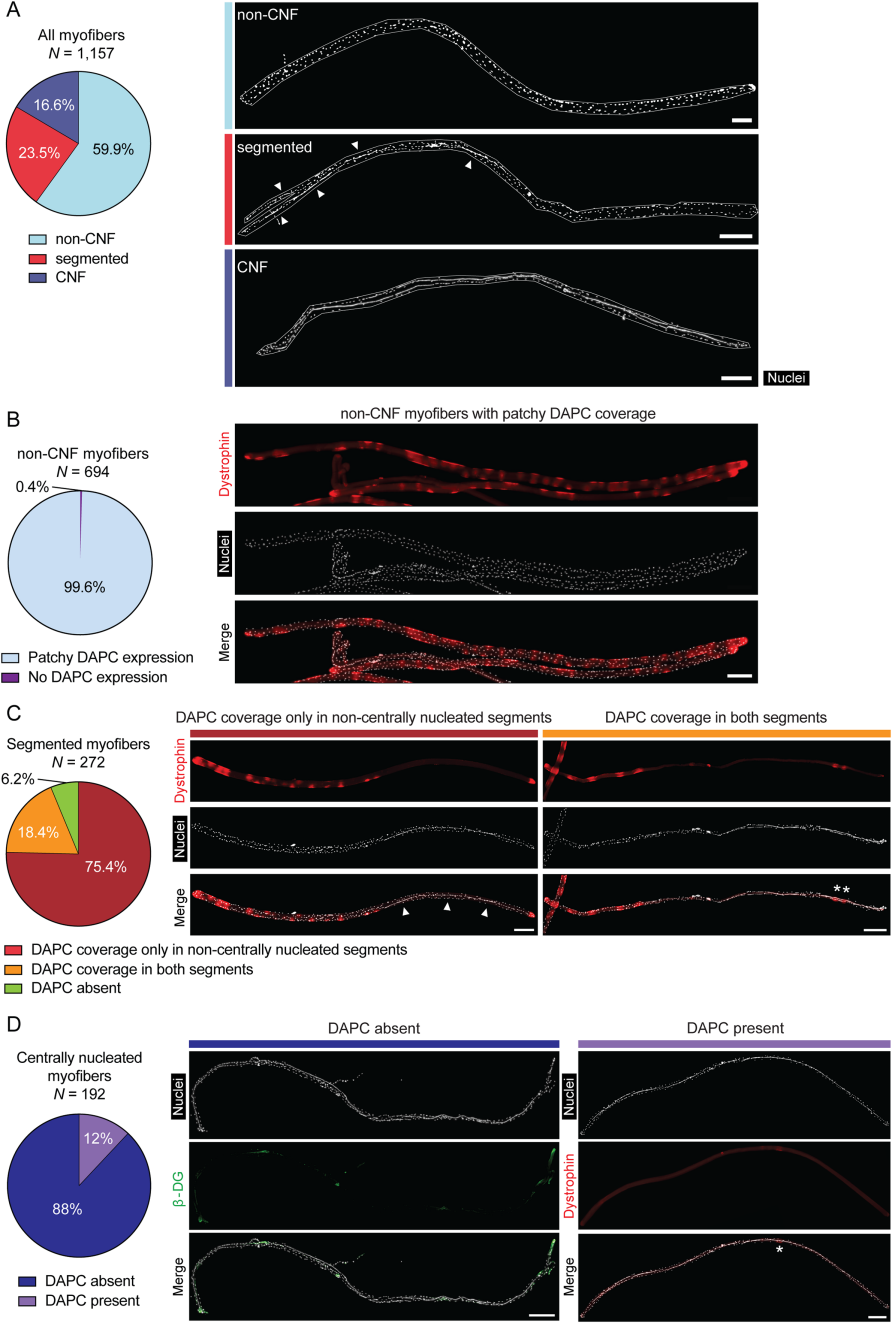
Dystrophin is largely absent in centrally nucleated myofibers/fiber segments. (A) Single isolated EDL myofibers harvested from adult (12–17 week‐old) *mdx52‐Xist* ^Δhs^ mice (*N* = 1 157) were sorted into noncentrally nucleated (non‐CNF), segmented (i.e., containing both centrally nucleated and noncentrally nucleated fiber regions), and fully centrally nucleated (CNF) categories. (B) Non‐CNFs (*N* = 694) were further classified into those with a ‘zebra‐like’ patchy DAPC pattern of expression, and those with no DAPC expression. (C) Segmented myofibers (*N* = 272) were further classified based on whether DAPC proteins (i.e., dystrophin or *β*‐dystroglycan) were detected in non‐CNF segments, both CNF and non‐CNF segments or absent from both segments. (D) Fully centrally nucleated myofibers (*N* = 192) were classified based on whether or not DAPC proteins were detected. Arrow heads indicate centrally nucleated regions of interest. Asterisks indicate DAPC‐positive regions of interest. Tiled images were acquired at 10× magnification and stitched together using the LAS X software. Scale bars indicate 200 μm. Nuclei were stained with DAPI.

Inspection of single isolated myofibers revealed the existence of three types of fiber based on the degree of central nucleation; (i) noncentrally nucleated (59.9%), (ii) uniformly centrally nucleated (16.6%) and (iii) segmented centrally nucleated, whereby chains of centrally located myonuclei were restricted to regions within the associated myofiber (23.5%) (Figure 5A). Noncentrally nucleated myofibers overwhelmingly (99.6%) exhibited patchy, ‘zebra‐like’ patterns of dystrophin distribution (Figure 5B, and similar to micrographs in Figures 2 and 4A). We next classified segmented fibers according to dystrophin/*β*‐dystroglycan (i.e., DAPC) expression, with signal in the noncentrally nucleated region only (75.4%), coverage in both segments (18.4%) or no dystrophin/DAPC expression present at all (6.2%) (Figure 5C). Similarly, centrally nucleated myofibers and myofiber segments were found to be almost completely devoid of dystrophin/*β*‐dystroglycan expression. In fully centrally nucleated myofibers, 88% contained no dystrophin or *β*‐dystroglycan (i.e., DAPC) expression. The remaining 12% of myofibers exhibited some expression, although this was frequently limited to very small regions of membrane (Figure 5D). Importantly, in DAPC‐positive fully centrally nucleated fibers, none exhibited the patchy, ‘zebra‐like’ staining pattern. This finding suggests that the observed dystrophin absence in centrally nucleated regions is very unlikely to be driven by an XCI effect associated with the *Xist* ^Δhs^ model.

**FIGURE 6:**
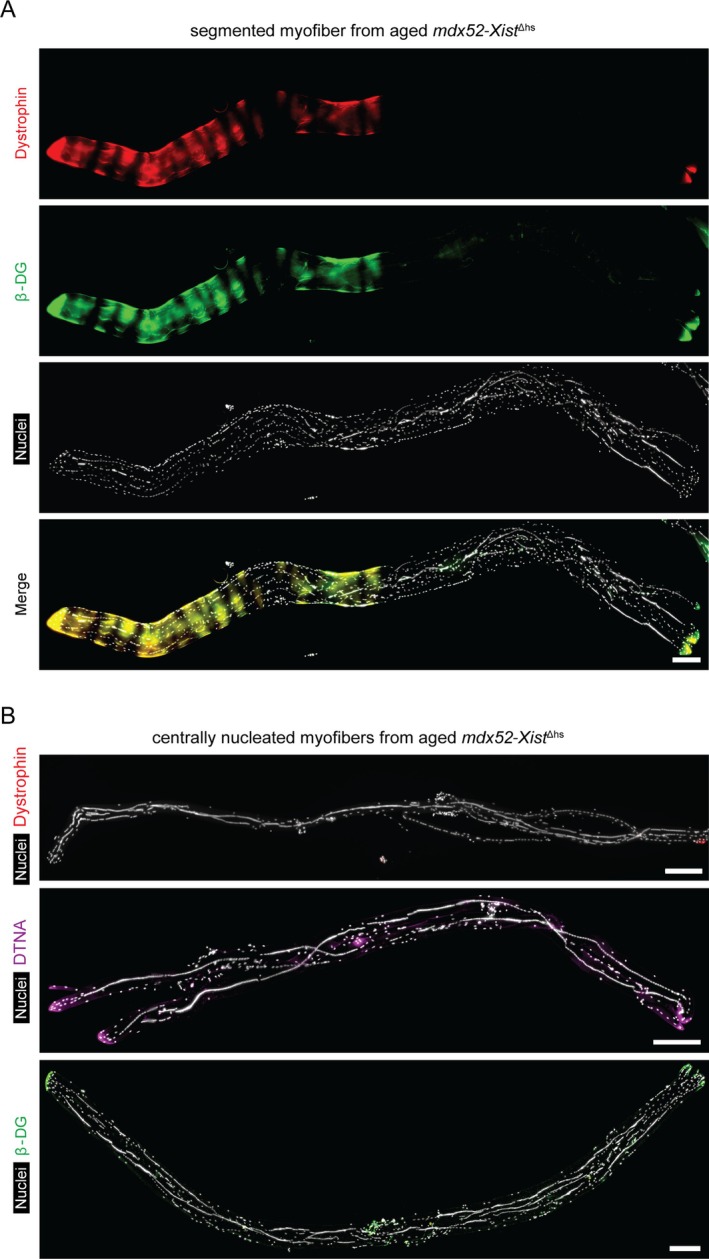
Dystrophin and DAPC protein expression is largely absent in centrally nucleated myofibers/fiber segments in aged *mdx52‐Xist* ^Δhs^ mice. Single EDL myofibers isolated from 60‐week‐old *mdx52‐Xist* ^Δhs^ mice were analysed by immunofluorescence and representative micrographs shown for (A) dystrophin and *β*‐dystroglycan (*β*‐DG) costaining in segmented myofibers (i.e., containing both centrally nucleated and noncentrally nucleated regions), and (B) dystrophin, *α*‐dystrobrevin (DTNA) and *β*‐DG in fully centrally nucleated myofibers. Tiled images were acquired at 10× magnification and stitched together using the LAS X software. Scale bars represent 200 μm. Nuclei were stained with DAPI.

The absence of dystrophin and *β*‐dystroglycan expression in centrally nucleated myofiber segments was even more apparent in segmented myofibers from aged (60‐week‐old) *mdx52‐Xist* ^Δhs^ animals (Figure 6A), with aged fully‐CNF myofibers being largely devoid of DAPC protein expression (Figure 6B). A representative bulk preparation of myofibers from a single 60‐week‐old *mdx52*‐*Xist* ^Δhs^ illustrating this point is shown in Figure S6. Notably, the aged myofibers were evidently hypertrophic and frequently contained multiple chains of centrally located myonuclei. These observations indicate that centrally nucleated myofibers in *mdx52‐Xist* ^Δhs^ mice at this age are not recently regenerated. Immunostaining for the nuclear envelope marker Lamin B1 (LAMB1) showed that these nuclei chains consist of intact myonuclei squashed together, rather than fused together (Figure S7). Muscle injury alone was insufficient to induce the dystrophin absence in CNF phenomenon (Figure S8). Taken together, these data show that dystrophin/the DAPC is largely absent in centrally nucleated myofibers and in the centrally nucleated regions of segmented myofibers. The absence of a similar effect in injured wild‐type muscle suggests that this phenomenon is a feature of dystrophic muscle.

### 3.5 Expression of Dystrophin Is Specifically Impaired at the Protein Level in Centrally Nucleated Myofiber Segments

**FIGURE 7:**
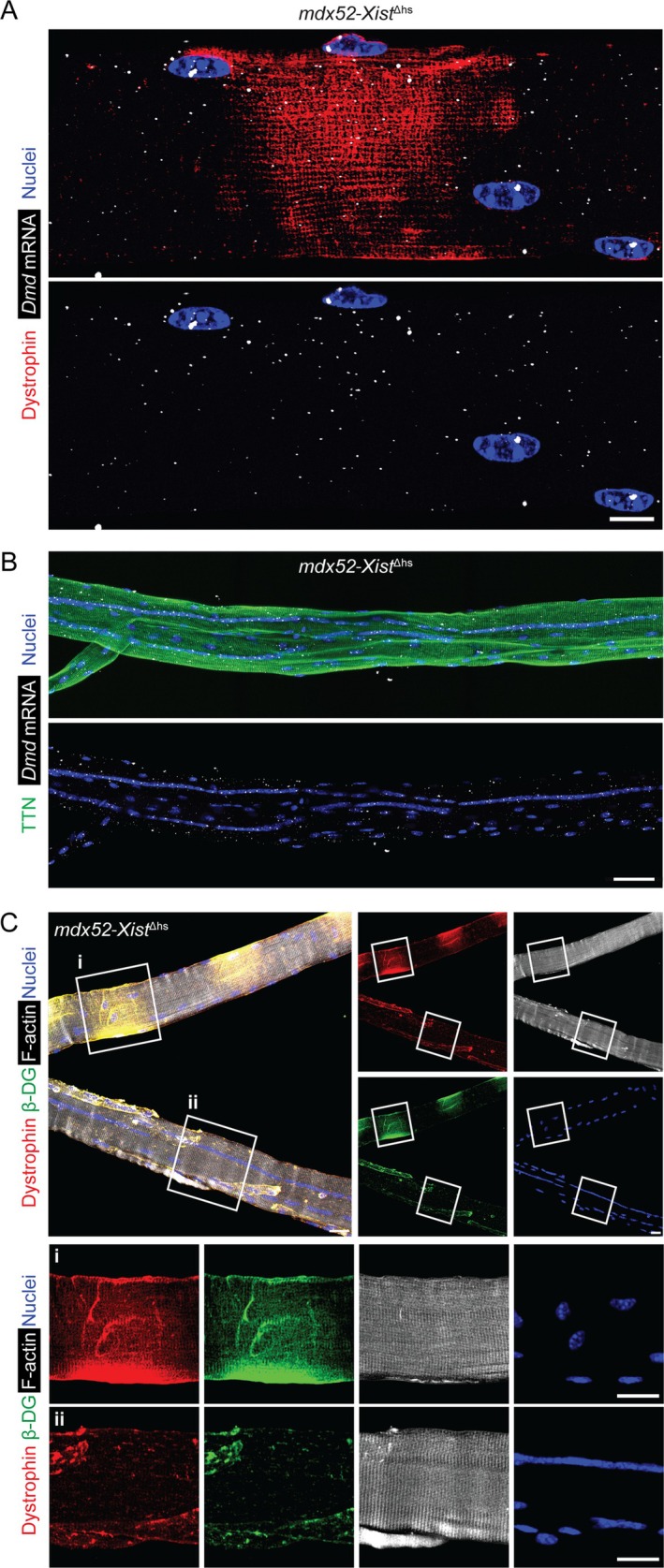
Dystrophin protein expression is suppressed in *mdx52‐Xist* ^Δhs^ centrally nucleated myofiber segments. Single EDL myofibers were isolated from adult *mdx52‐Xist* ^Δhs^ and analysed for immunofluorescence and RNA fluorescence in situ hybridization (FISH). (A) Representative micrograph of a single isolated EDL myofiber from an *mdx52‐Xist* ^Δhs^ animal (12‐week‐old) showing combined immunostaining for dystrophin protein and HCR‐FISH for *Dmd* mRNA. Image taken at 40× magnification, scale bar represents 10 μm. (B) Representative micrograph of a centrally nucleated myofiber stained for TTN protein and dystrophin mRNA. Tiled images were acquired at 25× magnification and stitched together using the ZEN Blue software. Scale bar represents 50 μm. (C) Representative micrograph of a segmented myofiber (i.e., containing both centrally nucleated and noncentrally nucleated regions) and a fully centrally nucleated myofiber in the same frame stained for dystrophin, *β*‐dystroglycan (*β*‐DG), and filamentous‐actin (F‐actin). Selected regions showing (i) a patchy, noncentrally nucleated segment and (ii) a centrally nucleated segment, are enlarged and shown inset. Images were acquired at 25 × magnification. Scale bars represent 20 μm. Nuclei were stained with DAPI.

The absence of dystrophin in centrally nucleated myofiber segments might be the result of a global impairment in either transcription or translation. RNA‐FISH analysis using a pool of probes spanning the *Dmd* transcript revealed puncta evenly distributed throughout *mdx52‐Xist* ^Δhs^ single isolated segmented myofibers independent of dystrophin expression (Figure 7A). (Notably, this assay is not capable of distinguishing between the mutant and wild‐type *Dmd* alleles). Analysis of centrally nucleated *mdx52‐Xist* ^Δhs^ single isolated myofibers showed that both *Dmd* transcripts and titin (TTN) protein were uniformly distributed throughout all myofibers assessed (Figure 7B). Moreover, TTN exhibited a characteristic pattern of sarcomeric striation, indicative of myofiber maturity. TTN and filamentous Actin (F‐actin) were found to be evenly distributed throughout both centrally nucleated and noncentrally nucleated myofiber regions, in stark contrast to the pattern observed for dystrophin (Figure 7C). These data demonstrate that there is no shortage or mislocalization of *Dmd* mRNAs in centrally nucleated myofiber regions, and that there is no local impairment in global protein translation. As such, these data suggest that dystrophin protein expression is specifically impaired in *mdx52‐Xist* ^Δhs^ centrally nucleated myofiber regions at the protein level.

### 3.6 Microtubule Network Disruption Is Similar in Dystrophin Positive and Negative Segments

**FIGURE 8:**
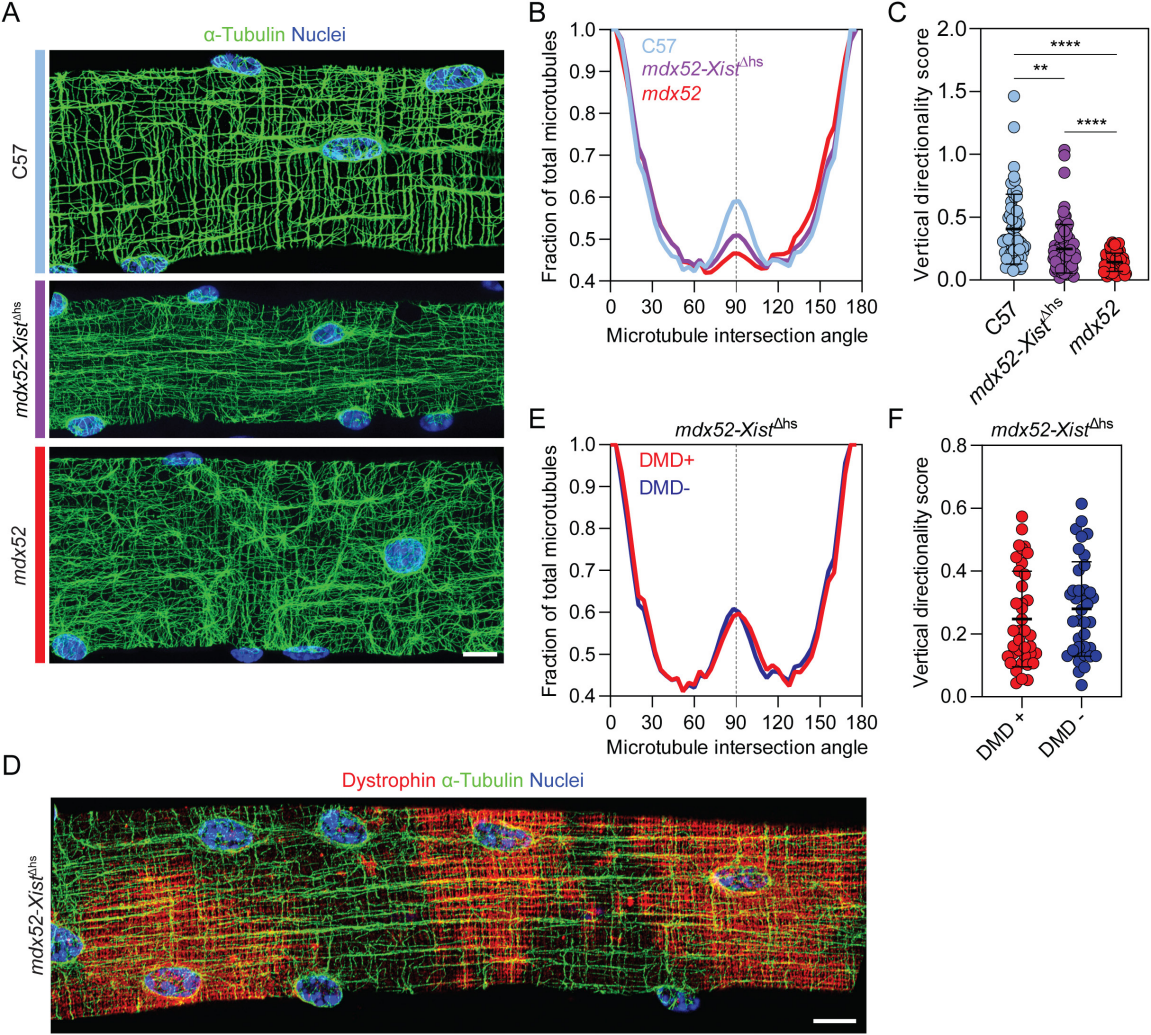
*mdx52‐Xist* ^Δhs^ myofibers exhibit intermediate microtubule network disorganization that is similar in dystrophin positive and negative segments. (A) Representative micrographs of immunostaining for *α*‐tubulin to show cortical microtubule network organization in adult (8–12 weeks old) C57 wild type, *mdx52* and *mdx52‐Xist* ^Δhs^ single isolated EDL myofibers. (B) Histogram of mean distribution of microtubules of different intersection angles relative to the myofiber long axis. The transverse, costameric microtubule peak (90°) is marked with a dotted line. (C) Vertical directionality scores reflecting the summed values of microtubules present between 80° to 100° within each myofiber. (Sample sizes are; C57: *n* = 60 ROIs, derived from 32 myofibers, *mdx52*: *n* = 64 ROIs, derived from 31 myofibers, *mdx52‐Xist* ^Δhs^: *n* = 79 ROIs, derived from 40 myofibers). (D) Representative micrographs of coimmunostaining for *α*‐tubulin and dystrophin to show cortical microtubule network organization in a patchy dystrophin *mdx52‐Xist* ^Δhs^ single isolated EDL myofiber. (E) Histogram of mean distribution of microtubules of different intersection angles relative to myofiber long axis in dystrophin positive and negative myonuclear domains of *mdx52‐Xist* ^Δhs^ EDL myofibers. The transverse, costameric microtubule peak (90°) is marked with a dotted line. (Sample sizes are; DMD+: *n* = 42 ROIs, derived from 24 myofibers, DMD‐: *n* = 40 ROIs, derived from 25 myofibers). (F) Vertical directionality score reflecting the summed values of microtubules present between 80° to 100° within each fiber. Images taken at 40× magnification, scale bars represent 10 μm. Plotted values are mean ± SD Statistically significant differences were assessed by one‐way ANOVA with Bonferroni post hoc test or two‐tailed Student's *t*‐test, as appropriate. **p < 0.01 and **** *p* < 0.0001. Nuclei were stained with DAPI.

The absence of dystrophin expression in centrally nucleated regions might be a consequence of impaired mRNA trafficking following microtubule network disruption. One of the hallmarks of correct myofiber organization is the intricately organized microtubule network, which was recently shown to facilitate the active transport of various RNAs and proteins, including the ribosomal machinery, throughout the cell [26, 27]. Notably, dystrophin protein contains a microtubule‐binding domain and therefore has been proposed to stabilize the myofiber microtubule cytoskeleton [28, 29]. This role is supported by the fact that the microtubule network is significantly disorganized in dystrophic mice, with costameric (transverse) components being the most severely affected [28, 30]. Thus, it is likely that nonuniformly distributed dystrophin can modify the organization of the microtubules in *mdx52‐Xist* ^Δhs^ mice, thereby partially facilitating correct mRNA transcript trafficking. TeDT (texture detection technique) analysis of microtubules was performed on images acquired from *mdx52‐Xist* ^Δhs^, *mdx52* and wild‐type C57 EDL myofibers (*n* = 40, 31 and 32 regions of interest, respectively). The characteristic peak at the 90° intersection angle (representing the transverse microtubules) together with a high vertical directionality score was detected in adult wild‐type C57 myofibers (Figure 8A–C). In agreement with previous reports, the microtubule network was visibly disorganized in *mdx52* animals, with a corresponding significant loss of transverse microtubules (Figure 8B,C) [28, 30]. This disorganized pattern was partially restored in *mdx52‐Xist* ^Δhs^ myofibers as represented by an intermediate distribution of microtubule intersection angles and vertical directionality scores (Figure 8B,C). These results show that nonuniformly distributed dystrophin in the *mdx52‐Xist* ^Δhs^ model is associated with an intermediately distorted microtubule network.

We were next motivated to determine whether there was a difference between microtubule network organization in dystrophin‐positive and ‐negative *mdx52‐Xist* ^Δhs^ myofiber segments (Figure 8D–F). No difference in microtubule lattice organization was observed between the analysed domains in terms of microtubule intersection angle distribution (Figure 8E) or vertical directionality scores (Figure 8F). This suggests that the local absence of dystrophin alone may not be sufficient to induce cytoskeletal network disruption.

### 3.7 Aged *mdx52‐Xist* ^Δhs^ Animals Contain High Proportions of Hypertrophic Centrally Nucleated Myofibers in the Absence of Active Regeneration

Central nucleation is associated with muscle regeneration, but is known to persist long after injury in mice (as long as 21 months) [31, 32]. In addition, regenerating myofibers exhibit small cross‐sectional areas and are positive for development‐associated markers such as embryonic myosin heavy chain and utrophin. To better understand the phenomenon of dystrophin absence in centrally nucleated myofibers/fiber segments, we analysed the extent of central nucleation in adult (6 week) and aged (60 week) *mdx52‐Xist* ^Δhs^ TA muscle sections.

Central nucleation was shown to significantly increase in TA muscle sections from aged animals (Figure S9A). Furthermore, there was a shift towards a greater number of central myonuclear chains with age in isolated EDL myofibers (Figure S9B). Analysis of myofiber cross‐sectional area revealed that aged *mdx52‐Xist* ^Δhs^ animals exhibited a pronounced shift towards larger fibers (Figure S9C), and that there was a statistically significant (*p* < 0.0001) shift in the mean Feret diameter in centrally nucleated fibers (Figure S9D). Taken together, these data show that CNFs undergo substantial hypertrophy with age, which is likely driven by the progressive accretion of new myonuclei. Indeed, the number of nuclei per myofiber volume was increased in CNF regions compared with non‐CNF regions in *mdx52* mice (Figure S10) [33, 34]. These observations, together with the limited dispersion of central nuclei to the myofiber periphery observed in mice [31, 32], demonstrate that CNFs at later stages largely do not constitute a population of recently formed muscle cells [32, 35]. Together, these results emphasize the fact that the proportion of CNF at later stages of life in mice reflects the cumulative history of regeneration, and not recently regenerating immature muscle [35]. The latter point is important, because myofiber immaturity could be a potential explanation for the absence of dystrophin in centrally nucleated myofiber regions. Furthermore, the expression of late‐stage markers of muscle maturity (i.e., TTN, Figure 7B) and the absence markers of early‐stage muscle development (i.e., UTRN, Figure S4) in CNFs lends further credence to the notion that these myofibers are not recently regenerating and immature.

Notably, in mature myofibers, nuclei play a crucial role as microtubule organization centres [36, 37]. As such, the distinct localization of myonuclei within CNFs and non‐CNFs could potentially affect the organization of the microtubule network. To assess the contribution of central nucleation to microtubule organization, cortical microtubules were analysed in CNF and non‐CNF EDL myofibers harvested from 12‐week‐old *mdx52* mice, as these muscles are expected to contain very high proportions of centrally nucleated myofibers [35]. The microtubule network was visibly disorganized in CNFs (Figure S9E), which was accompanied by a quantitative decrease in transverse microtubules (Figure S9F) and vertical directionality scores in CNFs (*p* < 0.0001) (Figure S9G). These results show that within dystrophic myofibers, the microtubule lattice is substantially more disrupted in centrally nucleated myofibers than in noncentrally nucleated myofibers.

## 4 Discussion

This study adds to the growing literature reporting spatial restriction of dystrophin protein to regions of sarcolemma proximal to their myonuclei of origin. Analyses in *mdx52‐Xist* ^Δhs^ mice revealed two distinct types of spatial phenomena. Firstly, a ‘zebra‐like’ patchy pattern of dystrophin was observed in the majority of myofibers, as is expected from the underlying pattern of skewed XCI of the healthy *Dmd* allele, and consistent with previous observations (Figures 1, 2, 3, 4, 5, 6, 7, 8) [9, 11, 14]. An increase in overall dystrophin protein expression levels was observed in aged vs. adult *mdx52‐Xist* ^Δhs^ animals (Figures 1C and 3C), consistent with the notion that newly dystrophin‐positive myofibers will tend to accumulate over time, as a consequence of a positive selection [4, 38]. Nevertheless, aged *mdx52‐Xist* ^Δhs^ animals still exhibited nonuniform patterns of sarcolemmal dystrophin, indicating that accumulation of dystrophin‐expressing regions is insufficient to completely resolve the observed sarcolemmal patchiness (Figures 3C and 4). These are disease‐relevant observations which mirror situations in which dystrophic myofibers may exist as heterokaryons containing both dystrophin‐expressing and nondystrophin‐expressing myonuclei, specifically in the case of female dystrophinopathy [22, 23], and in dystrophic muscle after partially‐effective CRISPR‐Cas9‐mediated dystrophin restoration [8, 9, 10, 11]. Importantly, other types of dystrophin‐restoration strategy (such as cell therapy) have the potential to generate myofiber heterokaryons, and consequently patchy sarcolemmal dystrophin coverage.

Concerning the second spatial phenomenon, we observed that dystrophin and dystrophin‐associated proteins were absent from centrally nucleated myofibers and myofiber segments (Figures 5, 6, 7, S6). This surprising finding suggests that centrally nucleated myofibers are refractory to dystrophin expression, at least in this model. Our first thought was that dystrophin may be absent as a consequence of myofiber immaturity [39]. However, several lines of evidence argue against this notion. Firstly, dystrophin absence in centrally nucleated regions was observed in 60‐week‐old mice (Figure 6, S6), when regeneration events are likely to be very limited (as also evidenced by limited staining for utrophin, a marker of regenerating myofibers, Figure S4). Secondly, *mdx52‐Xist* ^Δhs^ centrally nucleated fibers also exhibit; (i) sizes consistent with healthy (or hypertrophic) myofibers (Figures 8, S10), (ii) expression of late‐stage markers of muscle differentiation like TTN (Figures 7) and (iii) frequently contained multiple chains of central nuclei (Figures 6, 7, S6, S9 and S10). Together, these data suggest that these are in fact mature myofibers, exhibiting signs of repeated historical degeneration and accumulated repair.

A second possible explanation is that the absence of dystrophin in centrally nucleated myofibers and myofiber segments is simply reflecting the underlying XCI mosaicism. Specifically, that myofiber segments composed predominantly of nuclei with the inactive wild‐type X‐chromosome would be incapable of producing dystrophin and thereby be especially susceptible to myonecrosis, leading to the acquisition of central nuclei following regeneration. On this model, the absence of dystrophin leads to muscle turnover and central nucleation, rather than the absence of dystrophin being a consequence of the dystrophic environment in postregeneration fibers. For the absence of dystrophin in centrally nucleated myofiber phenomenon to be spatially stable, the affected myofiber segments would need to lack any neighbouring satellite cells capable of expressing dystrophin. Given the mixed populations of satellite cells (both dystrophin‐competent and dystrophin‐deficient) surrounding each fiber in three dimensions, repeated regenerative events would be expected to repopulate such segments with at least some dystrophin‐competent myonuclei. Instead, we never observe centrally nucleated myofibers that exhibit the ‘zebra‐like’ patchy pattern of dystrophin expression (Figures 5, S6).

This XCI‐based explanation necessitates the existence of very extreme skews in the distribution of cells according to their capability of expressing dystrophin. It follows that if there are large clusters of dystrophin‐incompetent myonuclei (leading to uniformly dystrophin absent centrally nucleated regions), then statistically we would also expect to observe large clusters composed of dystrophin‐capable myonuclei (leading to noncentrally nucleated regions uniformly expressing dystrophin), which we do not observe (Figure 5, S6). Furthermore, the accretion of dystrophin‐expressing myonuclei into centrally nucleated regenerated regions might be expected to provide a selection advantage, leading to subsequent myofiber stabilization and enrichment over time, making the absence of dystrophin‐expressing, postregeneration centrally nucleated myofiber segments particularly notable. In reality, it is highly likely that all myofibers contain mixtures of dystrophin‐competent and ‐incompetent myonuclei. As such, it is unlikely that central nucleation‐associated absence of dystrophin expression can be explained by model‐associated XCI effects alone. The presence of dystrophin protein in the postregeneration muscles in wild‐type mice (Figure S8) suggests that this phenomenon is associated with the dystrophic condition, and not regeneration *per se*.

We propose that dystrophin is specifically repressed at the protein level based on the uniform distribution of *Dmd* mRNA and TTN/F‐actin protein expression across both centrally nucleated and noncentrally nucleated *mdx52*‐*Xist* ^Δhs^ myofiber regions (Figure 7). Further work is needed to determine the mechanism of this repression, which may be the result of a primary failure in translation, or a posttranslational reduction in protein stability. It is tempting to speculate that local accumulation of *trans*‐acting factors, such as miRNAs, may be responsible. For example, miR‐31 has been reported to repress dystrophin expression and is upregulated in dystrophic muscle [40].

The absence of dystrophin in centrally nucleated myofibers/fiber‐segments is a disease‐relevant observation, as it is suggestive of an additional challenge to the successful reintroduction of dystrophin protein in dystrophic muscle, which may limit the effectiveness of current and future experimental therapeutic interventions. Indeed, such a discrepancy between RNA‐level exon skipping levels and dystrophin protein levels after antisense oligonucleotide treatment in DMD patients has been previously reported [41]. Importantly, we have previously reported widespread rescue of dystrophin expression after antisense oligonucleotide‐mediated exon skipping with highly potent PPMO compounds [8, 9], which would appear to contradict with the findings reported herein. Notably, treated animals are typically analysed using transverse muscle sections (with isolated single myofiber analysis being relatively rare). As such, some within‐fiber patchiness may have been obscured in these analyses. Alternatively, high levels of exon skipping may have been sufficient to overcome the mechanism that represses dystrophin protein expression in centrally nucleated fibers (i.e., there is a threshold effect).

The *mdx52*‐*Xist* ^Δhs^ model presents a unique opportunity to study dystrophin‐dependent and CNF‐associated spatial phenomena in myofiber heterokaryons. However, it remains to be determined if such effects are present in DMD patient muscle. Dystrophin patchiness has been reported in female dystrophinopathy, and Torelli et al. have reported an inverse relationship between differential sarcoplasmic dystrophin coverage and disease severity in patient biopsies [12]. Importantly, centrally located myonuclei are known to migrate to the myofiber periphery following the completion of regeneration in human muscle, in contrast with the situation in mouse [31, 32]. However, experimentally determining whether central nucleation‐associated impairment in dystrophin expression similarly occurs in human muscle may be challenging.

In conclusion, this work has identified a previously unappreciated level of subcellular complexity in gene expression regulation in skeletal muscle with important implications for efforts to restore dystrophin protein expression in the muscles of DMD patients.

## Conflicts of Interest

M.J.A.W. discloses being an advisor and shareholder in PepGen Ltd., a biotechnology company that aims to generate exon skipping therapies for DMD. M.J.A.W. has filed multiple patents relating to exon skipping technologies for treating DMD. A.A.R. discloses being employed by LUMC, which has patents on exon skipping technology, some of which have been licensed to BioMarin and subsequently sublicensed to Sarepta. As coinventor of some of these patents, A.A.R. was entitled to a share of royalties. A.A.R. further discloses being an ad hoc consultant for PTC Therapeutics, Sarepta Therapeutics, Regenxbio, Dyne Therapeutics, Lilly, BioMarin Pharmaceuticals Inc., Eisai, Entrada, Takeda, Splicesense, Galapagos, Sapreme, Italfarmaco and Astra Zeneca. A.A.R. also reports being a member of the scientific advisory boards of Hybridize Therapeutics (past), Silence Therapeutics, Sarepta Therapeutics, Sapreme and Mitorx. Remuneration for consulting and advising activities is paid to LUMC. In the past 5 years, LUMC also received speaker honoraria from Alnylam Netherlands, Italfarmaco, and Pfizer, and funding for contract research from Sapreme, Eisai, BioMarin, Galapagos and Synaffix. Project funding is received from Sarepta Therapeutics and Entrada via unrestricted grants. R.J.P. has received funding for separate research programmes from Pfizer, Ultragenyx, and Exonics Therapeutics and has been a consultant to Exonics Therapeutics; the financial interests were reviewed and approved by the University in accordance with conflict of interest policies. The remaining authors declare no competing financial interests.

## Supporting information


**Figure S1:** Breeding scheme for *mdx52‐Xist*Δhs mice.
**Figure S2:** Dystrophin expression is inversely correlated with histopathology in *mdx52‐Xist*Δhs muscle sections.
**Figure S3:** Dystrophin expression is inversely correlated with histopathology in aged *mdx52‐Xist*Δhs muscle sections.
**Figure S4:** Utrophin expression is associated with muscle regeneration and is not reciprocal with dystrophin expression.
**Figure S5:** Analysis of myomiR biomarkers in adult and aged *mdx52‐Xist*Δhs serum.
**Figure S6:** Bulk *mdx52‐Xist*Δhs myofiber preparations illustrate two spatial dystrophin expression phenomena.
**Figure S7:** Myonuclei in centrally nucleated fibers are discrete and not fused.
**Figure S8:** Postregeneration, centrally nucleated myofibers express dystrophin in wild‐type mice.
**Figure S9:** Central nucleation accumulates with age in *mdx52‐Xist*Δhs mice and is associated with microtubule network disruption.
**Figure S10:** Nuclei numbers are increased in *mdx52* centrally nucleated myofiber segments.
**Figure S11:** Schema of single myofiber classification.
**Table S1:** Primary antibodies used in this study.
**Table S2:** Secondary antibodies used in this study.
**Table S3:** List of Small RNA TaqMan assays used in this study.

## Data Availability

All data are included in the manuscript. Raw data are available on request.
