# Supplementary material for: Myonuclear Domain‐Associated and Central Nucleation‐Dependent Spatial Restriction of Dystrophin Protein Expression
Source: J Cachexia Sarcopenia Muscle. 2026 Apr 7;17(2):e70284. doi: 10.1002/jcsm.70284 (PMC13055465; doi:10.1002/jcsm.70284)
Supplement: Supplementary file 1 — Figure S1: Breeding scheme for mdx52‐XistΔhs mice. Figure S2: Dystrophin expression is inversely correlated with histopathology in mdx52‐XistΔhs muscle sections. Figure S3: Dystrophin expression is inversely correlated with histopathology in aged mdx52‐XistΔhs muscle sections. Figure S4: Utrophin expression is associated with muscle regeneration and is not reciprocal with dystrophin expression. Figure S5: Analysis of myomiR biomarkers in adult and aged mdx52‐XistΔhs serum. Figure S6: Bulk mdx52‐XistΔhs myofiber preparations illustrate two spatial dystrophin expression phenomena. Figure S7: Myonuclei in centrally nucleated fibers are discrete and not fused. Figure S8: Postregeneration, centrally nucleated myofibers express dystrophin in wild‐type mice. Figure S9: Central nucleation accumulates with age in mdx52‐XistΔhs mice and is associated with microtubule network disruption. Figure S10: Nuclei numbers are increased in mdx52 centrally nucleated myofiber segments. Figure S11: Schema of single myofiber classification. Table S1: Primary antibodies used in this study. Table S2: Secondary antibodies used in this study. Table S3: List of Small RNA TaqMan assays used in this study. [file JCSM-17-e70284-s001.pdf]

Supplementary Information

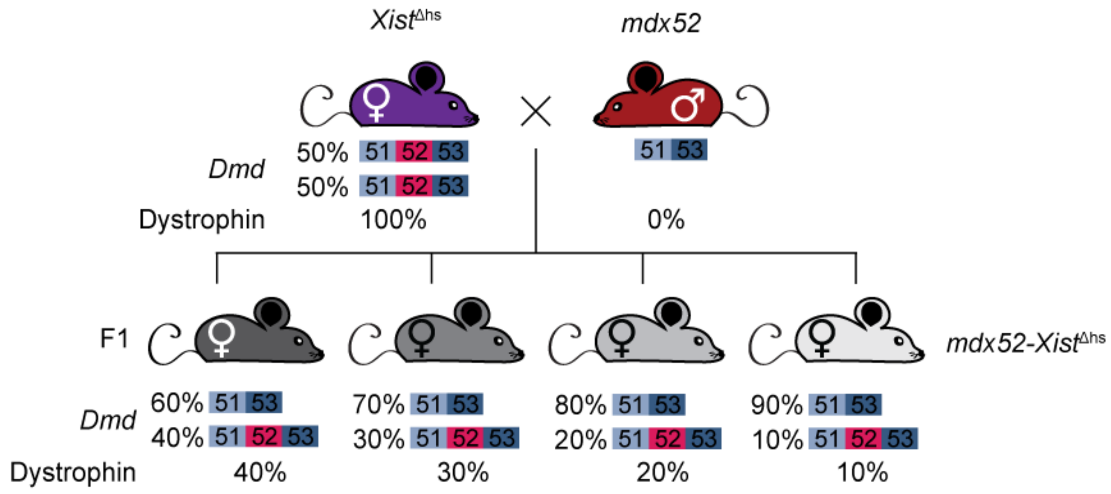

Figure S1

Breeding scheme for *mdx52-Xist*$^{\Delta hs}$ mice.

Breeding scheme to generate the *mdx52-Xist*$^{\Delta hs}$ model. Female *Xist*$^{\Delta hs}$ mice (containing a deletion in a DNase I hypersensitivity site in the *Xist* promoter) were crossed with male *mdx52* animals (lacking exon 52 of the *Dmd* gene). The resulting female F1 generation (*mdx52-Xist*$^{\Delta hs}$) are expected to exhibit variable levels of dystrophin expression as a consequence of skewed X-chromosome inactivation of the healthy *Dmd* allele. Examples of hypothetical dystrophin expression outcomes are shown (i.e. silencing of the healthy *Dmd*-containing chromosome in 60-90% of cases). For each mouse, the expected X-chromosome inactivation proportions and dystrophin protein expression are indicated.

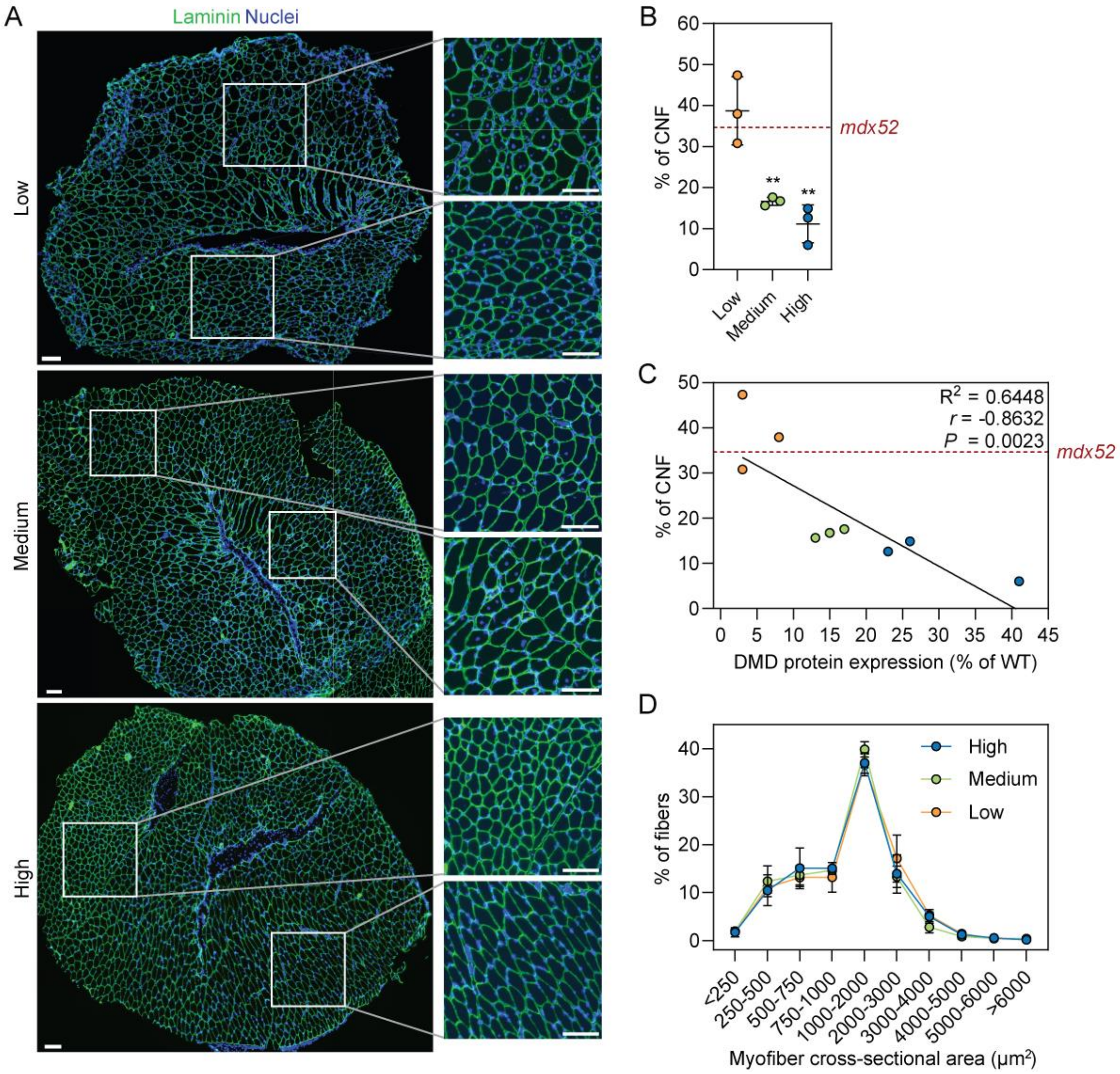
A
Laminin Nuclei
Low
Medium
High
B
% of CNF
mdx52
Low
Medium
High
C
R² = 0.6448
r = -0.8632
P = 0.0023
% of CNF
mdx52
DMD protein expression (% of WT)
D
% of fibers
High
Medium
Low
Myofiber cross-sectional area (µm²)
<250
250-500
500-750
750-1000
1000-2000
2000-3000
3000-4000
4000-5000
5000-6000
>6000

Figure S2

Dystrophin expression is inversely correlated with histopathology in *mdx52-Xist*$^{\Delta hs}$ muscle sections.

(**A**) Representative immunofluorescence images of transverse TA muscle sections from adult (6-week-old) *mdx52-Xist*$^{\Delta hs}$ stained for laminin (green) as a sarcolemma marker and DAPI (blue) for nuclei visualisation. A magnified view of the sections shows the centrally nucleated fibers (CNF) proportion in two regions of the section. Tiled images were taken at 10× magnification and stitched together using the LAS X software. Scale bars represent 100 µm. (**B**) CNF as a proportion of total myofibers analysed per TA section from *mdx52-Xist*$^{\Delta hs}$ animals expressing low, medium, and high dystrophin levels (n=3). A single *mdx52* animal was included as a reference. (**C**) Correlation analysis of dystrophin percentage and average CNF percentage in *mdx52-Xist*$^{\Delta hs}$ TA sections, with a single *mdx52* animal as a reference. Dystrophin protein percentage was derived from western blot analysis. (**D**) Myofiber size variability in TA sections of *mdx52-Xist*$^{\Delta hs}$ animals (n=3) visualized through proportion of fibers of specific cross-sectional area (CSA). Values are mean±SD. Statistical significance was assessed by one-way ANOVA with Bonferroni *post hoc* test relative to the Low dystrophin expressing group, $^{**}P<0.01$. Nuclei were stained with DAPI.

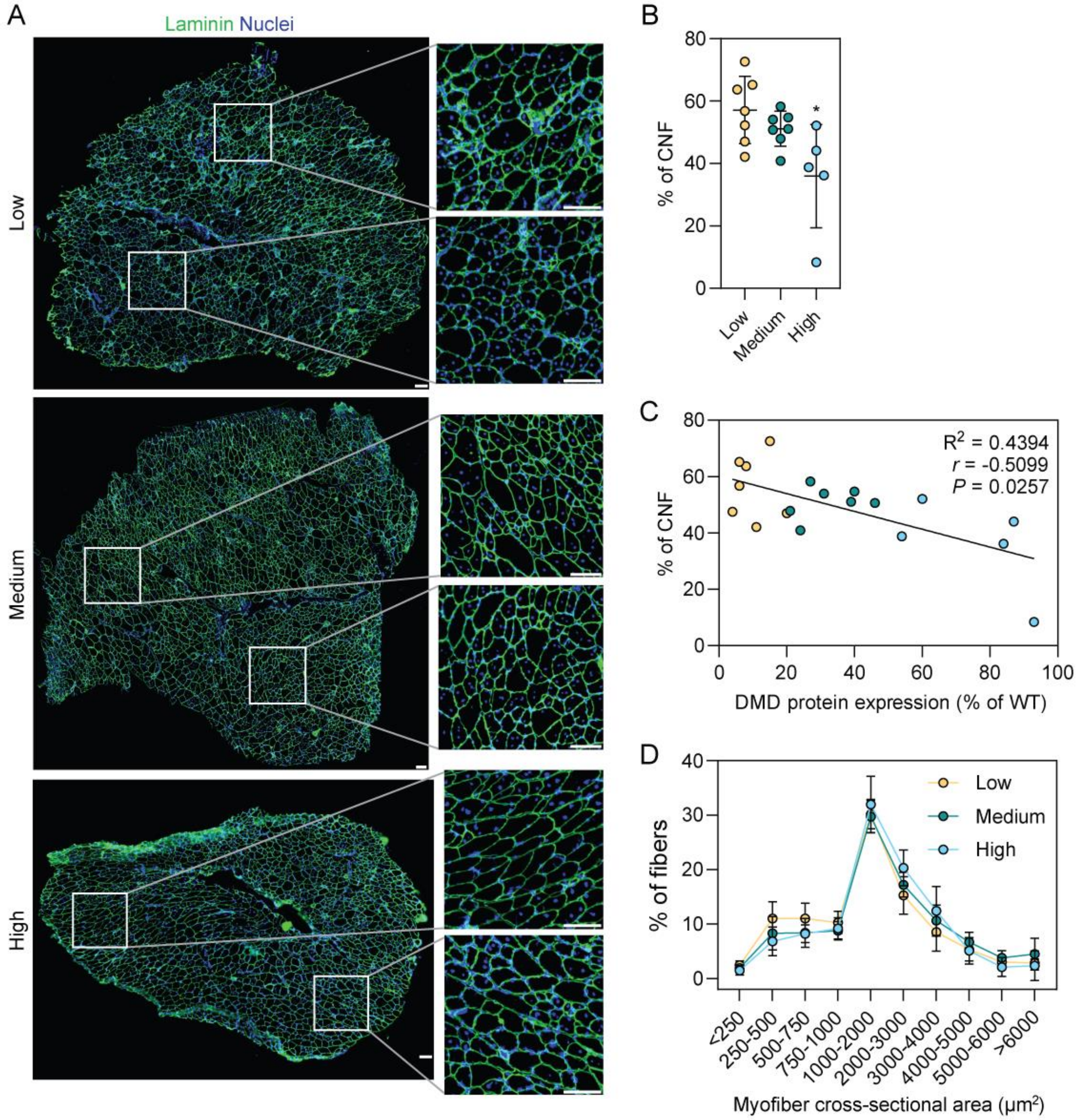
A
Laminin Nuclei
Low
Medium
High
B
% of CNF
Low
Medium
High
C
R^2 = 0.4394
r = -0.5099
P = 0.0257
% of CNF
DMD protein expression (% of WT)
D
% of fibers
Low
Medium
High
<250
250-500
500-750
750-1000
1000-2000
2000-3000
3000-4000
4000-5000
5000-6000
>6000
Myofiber cross-sectional area (μm^2)

Figure S3

Dystrophin expression is inversely correlated with histopathology in aged *mdx52-Xist*$^{\Delta hs}$ muscle sections.

(**A**) Representative immunofluorescence images of transverse TA muscle sections stained from aged (60-week-old) *mdx52-Xist*$^{\Delta hs}$ animals expressing high (*n*=5), medium (*n*=7), and low (*n*=7) dystrophin levels. Sections were stained for laminin (green) as a sarcolemma marker and DAPI (blue) for nuclei visualisation. A magnified view of the sections shows the centrally nucleated fibers (CNF) proportion in two regions of the section. Tiled images were taken at 10× magnification and stitched together using the LAS X software. Scale bars represent 100 µm. (**B**) CNF as a proportion of total myofibers analysed per TA section. (**C**) Correlation analysis of dystrophin percentage and average CNF percentage in analysed *mdx52-Xist*$^{\Delta hs}$ TA sections. (**D**) Myofiber size variability in TA sections of *mdx52-Xist*$^{\Delta hs}$ animals visualised through proportion of fibers of specific cross-sectional area (CNS). Values are mean±SD. Statistical significance was assessed by one-way ANOVA with Bonferroni *post hoc* test relative to the Low dystrophin expressing group, *P<0.05. Nuclei were stained with DAPI.

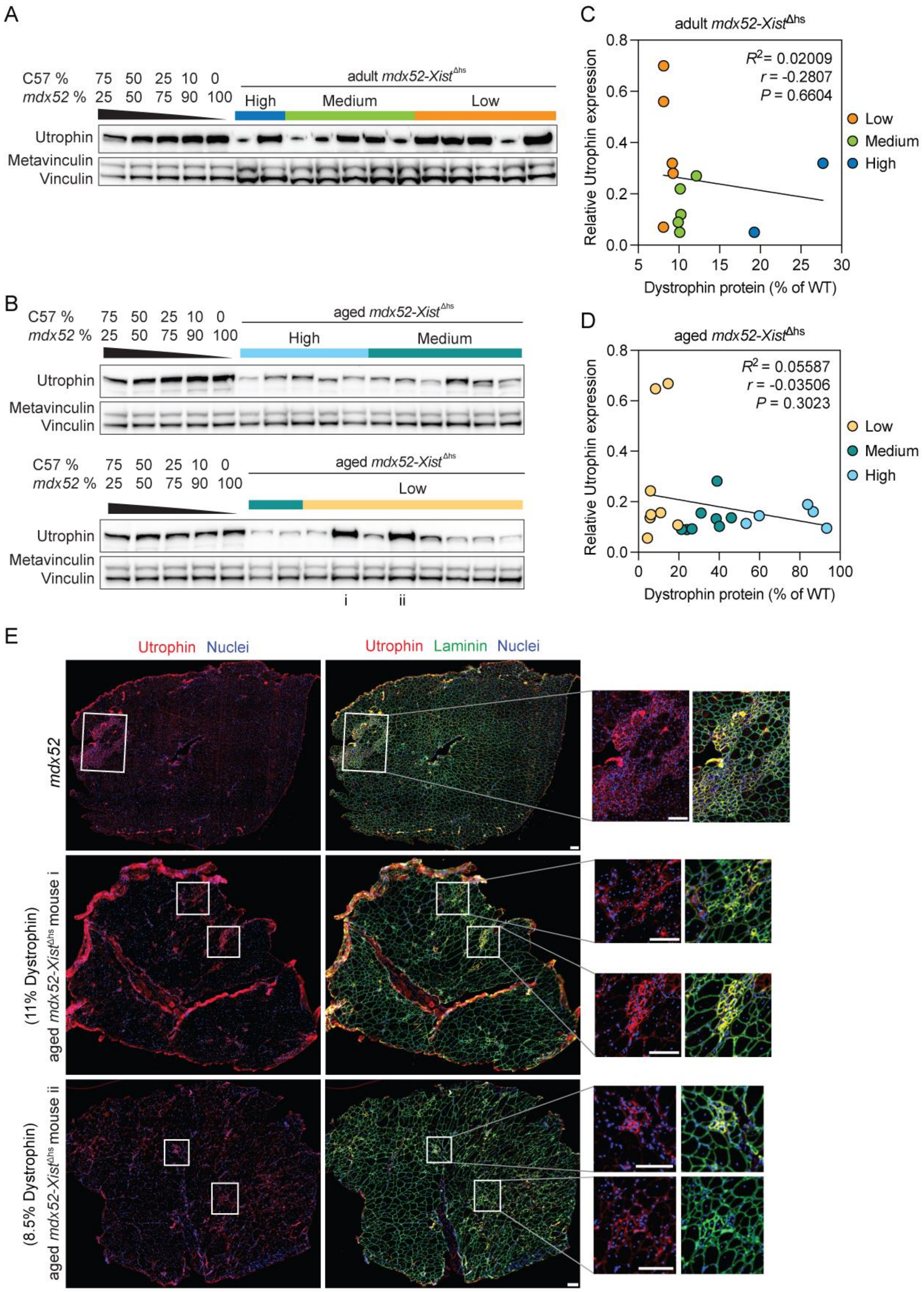
A
C57 % 75 50 25 10 0
mdx52 % 25 50 75 90 100
adult mdx52-Xist^Δhs
High Medium Low
Utrophin
Metavinculin
Vinculin
B
aged mdx52-Xist^Δhs
High Medium
Low
i ii
C
adult mdx52-Xist^Δhs
R^2= 0.02009
r = -0.2807
P = 0.6604
Relative Utrophin expression
Dystrophin protein (% of WT)
D
aged mdx52-Xist^Δhs
R^2 = 0.05587
r = -0.03506
P = 0.3023
E
Utrophin Nuclei
Utrophin Laminin Nuclei
mdx52
(11% Dystrophin) aged mdx52-Xist^Δhs mouse i
(8.5% Dystrophin) aged mdx52-Xist^Δhs mouse ii

Figure S4

Utrophin expression is associated with muscle regeneration and is not reciprocal with dystrophin expression.

Western blot analysis of utrophin protein in TA muscle from (**A**) adult (6 weeks old) and (**B**) aged (60 weeks old) *mdx52-Xist*$^{\Delta hs}$ mice. Vinculin was utilised as a loading control. Labelled, coloured boxes demonstrate levels of dystrophin expressed in respective samples. Spearman correlation analysis of dystrophin percentage and relative utrophin abundance in TA muscles of (**C**) adult and (**D**) aged *mdx52-Xist*$^{\Delta hs}$ animals. (**E**) Immunofluorescence staining of utrophin and laminin in TA muscle sections of 12-week-old *mdx52* mouse and 60-week-old *mdx52-Xist*$^{\Delta hs}$ animals from low dystrophin group expressing high levels of utrophin as quantified by western blot. Specific mice with high utrophin expression were selected for immunofluorescence analysis and are labelled as 'i' and 'ii' on the western blot. Insets demonstrate utrophin signal detected in small-calibre centrally nucleated myofibers in control *mdx52* and both *mdx52-Xist*$^{\Delta hs}$ animals. Values are mean±SD, *n*=2-8. Scale bars represent 100 µm, tiled images taken at 20× magnification and stitched together using LAS X.

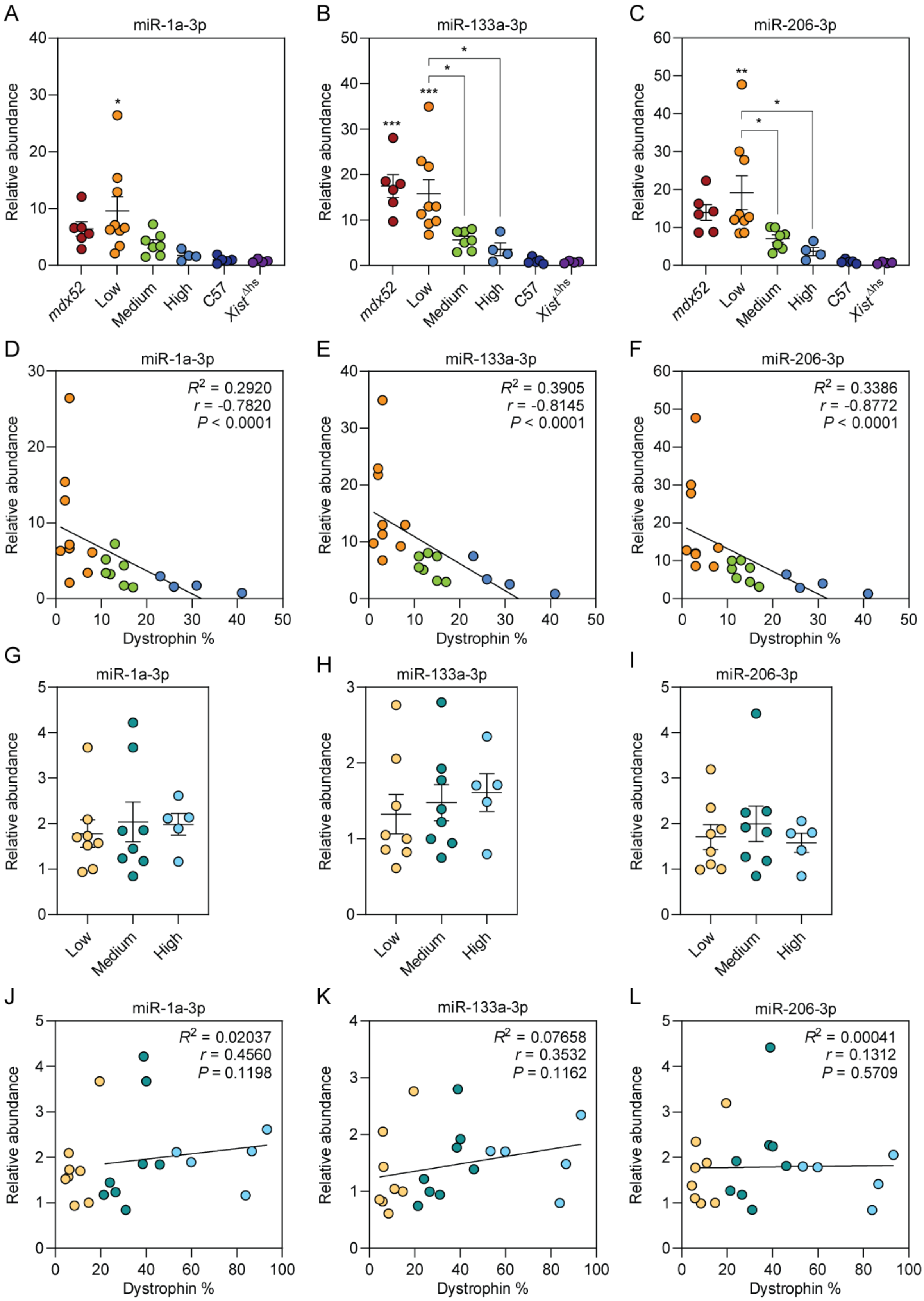
A
miR-1a-3p
Relative abundance
mdx52
Low
Medium
High
C57
Xist$^{\Delta hs}$
B
miR-133a-3p
C
miR-206-3p
D
R^2 = 0.2920
r = -0.7820
P < 0.0001
Dystrophin %
E
R^2 = 0.3905
r = -0.8145
P < 0.0001
F
R^2 = 0.3386
r = -0.8772
P < 0.0001
G
H
I
J
R^2 = 0.02037
r = 0.4560
P = 0.1198
K
R^2 = 0.07658
r = 0.3532
P = 0.1162
L
R^2 = 0.00041
r = 0.1312
P = 0.5709

Figure S5

Analysis of myomiR biomarkers in adult and aged *mdx52-Xist*$^{\Delta hs}$ serum.

Serum myomiR levels were analysed in adult (6-week-old) *mdx52-Xist*$^{\Delta hs}$ mice and compared with age and sex-matched dystrophic *mdx52*, and wild-type C57 and *Xist*$^{\Delta hs}$ controls for (**A**) miR-1a-3p, (**B**), miR-133a-3p, and (**C**) miR-206-3p. For each miRNA, the relationship between serum abundance and dystrophin protein expression in TA was analysed by Spearman correlation and linear regression (**D**-**F**). Serum myomiR levels were also analysed in aged (60-week-old) *mdx52-Xist*$^{\Delta hs}$ mice for (**G**) miR-1a-3p, (**H**), miR-133a-3p, and (**I**) miR-206-3p, and correlated with dystrophin protein expression in TA as above (**J**-**L**) Statistical significance was assessed by one-way ANOVA and Bonferroni *post hoc* test. Values are mean±SEM, *n*=4-9. *P<0.05, **P<0.01, ***P<0.001. Statistical comparisons are to the C57 wild-type group unless otherwise indicated.

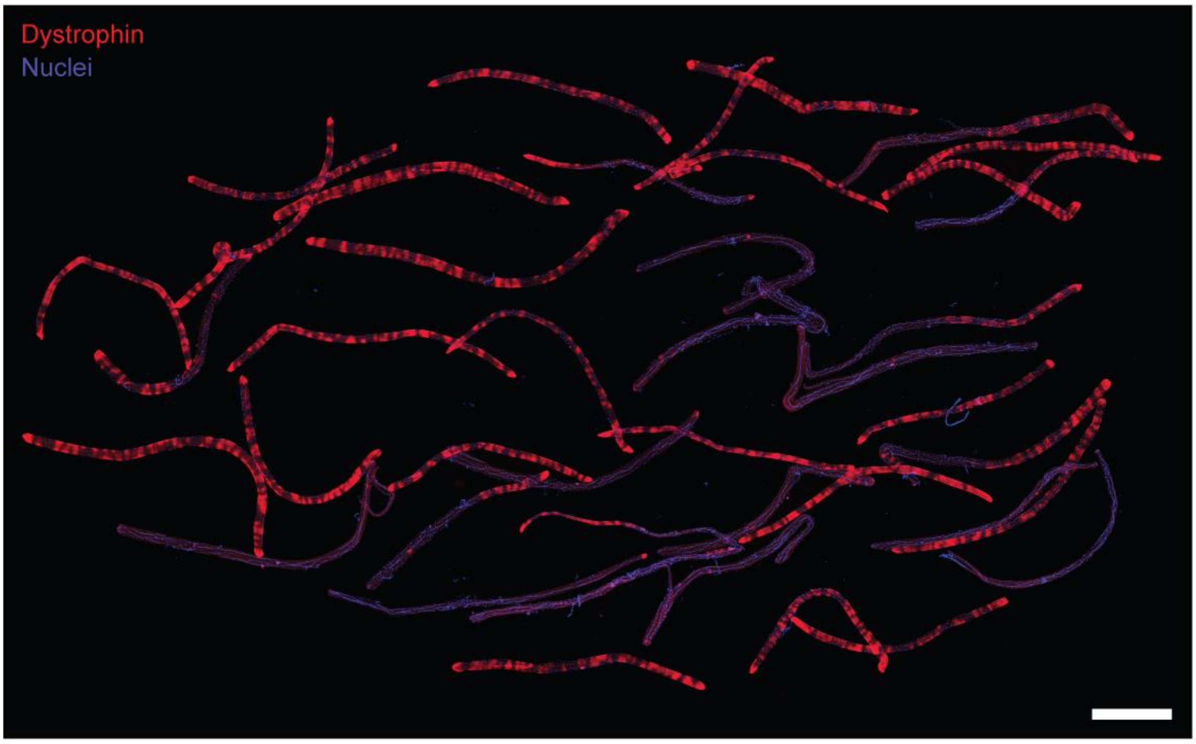

Figure S6

Bulk *mdx52-Xist*$^{\Delta hs}$ myofiber preparations illustrate two spatial dystrophin expression phenomena.

Composite micrograph showing dystrophin immunostaining in a bulk preparation of EDL myofibers isolated from a single 60-week-old *mdx52-Xist*$^{\Delta hs}$ animal. Scale bars represent 1,000 µm, tiled images taken at 20× magnification and stitched together using LAS X. Nuclei were stained with DAPI. Both spatial dystrophin expression phenomena are apparent in this micrograph; (i) a 'zebra-like' banding pattern of patchy dystrophin expression, and (ii) the absence of dystrophin expression in centrally nucleated myofibers and myofiber segments.

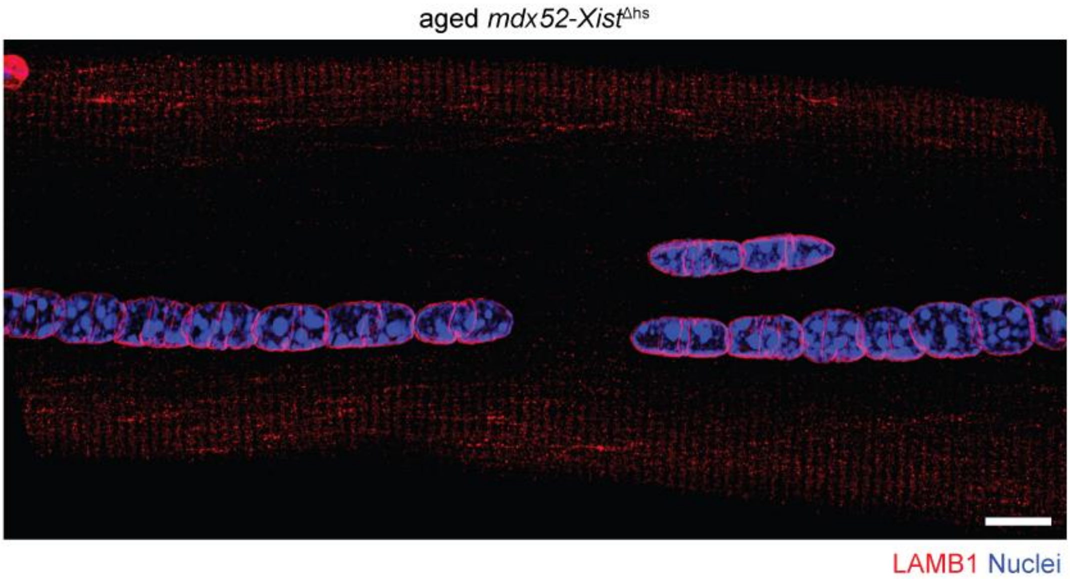

Figure S7

Myonuclei in centrally nucleated fibers are discrete and not fused.

Representative micrographs of immunostaining for LAMB1 (lamin-B1) to show nuclear membrane organization in single isolated EDL myofibers from 60-week-old *mdx52-Xist*$^{\Delta hs}$ mice. The staining shows that the myonuclei exhibit discrete nuclear membranes suggesting that they are packed closely together, rather than being fused. Images taken at 40× magnification, scale bar represents 10 µm.

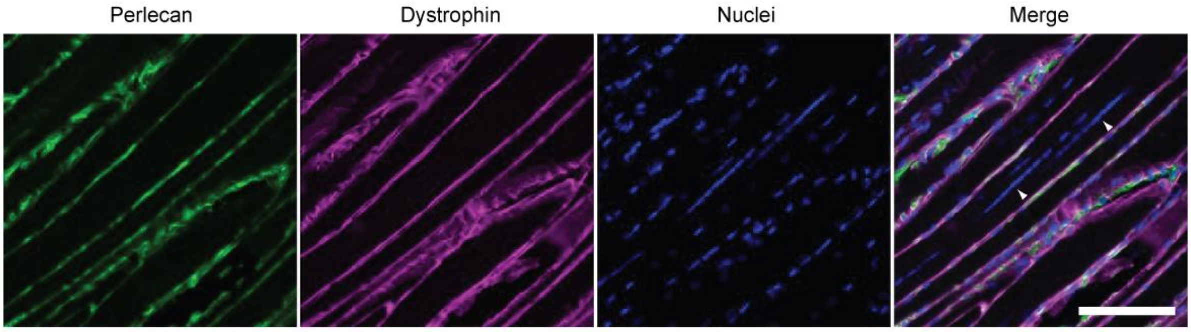

Figure S8

Post-regeneration, centrally nucleated myofibers express dystrophin in wild-type mice.

Representative micrographs showing a longitudinal section through the tibialis anterior muscle of a $BaCl_2$-treated wild-type mouse (29 days post injury). The basement membrane was stained using antibodies against Perlecan, and nuclei were stained using Hoechst. Regions of centrally located nuclei is highlighted with arrowheads. Images taken at 20× magnification, scale bar represents 100 µm.

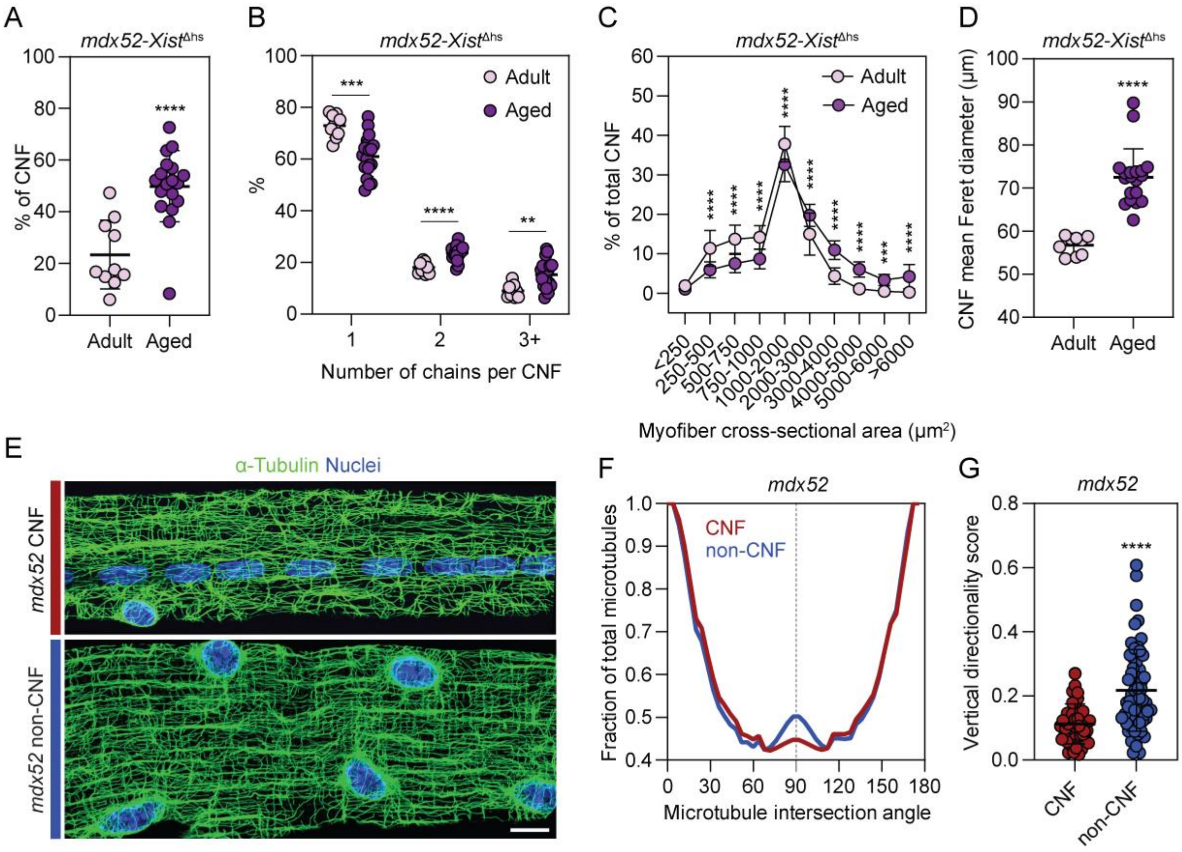
A
mdx52-Xist^Δhs
% of CNF
Adult
Aged
B
Number of chains per CNF
C
% of total CNF
Myofiber cross-sectional area (μm²)
D
CNF mean Feret diameter (μm)
E
α-Tubulin Nuclei
mdx52 CNF
mdx52 non-CNF
F
mdx52
Fraction of total microtubules
Microtubule intersection angle
CNF
non-CNF
G
Vertical directionality score

Figure S9

Central nucleation accumulates with age in *mdx52-Xist*$^{\Delta hs}$ mice and is associated with microtubule network disruption.

Adult (6-week-old) and aged (60-week-old) *mdx52-Xist*$^{\Delta hs}$ TA muscle sections were compared for (**A**) the percentage of CNF myofibers, (**B**) the number of chains per centrally nucleated myofiber, (**C**) mean Feret diameter of CNFs, and (**D**) Distribution of CNF cross-sectional area (CSA). Separately, adult (12-week-old) *mdx52* single isolated EDL myofibers were harvested and analysed for microtubule network organization in centrally nucleated and non-centrally nucleated myofibers. (**E**) Representative micrographs of immunostaining for α-tubulin. (**F**) Histogram of mean distribution of microtubules of different intersection angles relative to myofiber long axis in centrally nucleated and non-centrally nucleated *mdx52* myofibers. The transverse, costameric microtubule peak (90°) is marked with a dotted line. (**G**) Vertical directionality score reflecting the summed values of microtubules present between 80 to 100 degrees within each fiber. (Sample sizes are; CNF: n=44 ROIs, derived from 22 myofibers, non-CNF: n=60 ROIs, derived from 27 myofibers). Images taken at 40× magnification, scale bar represents 10 µm. Values are mean±SD. Statistically significant differences were assessed by Student's t-test, $^{**}P<0.01$, $^{***}P<0.001$, $^{****}P<0.0001$. Nuclei were stained with DAPI.

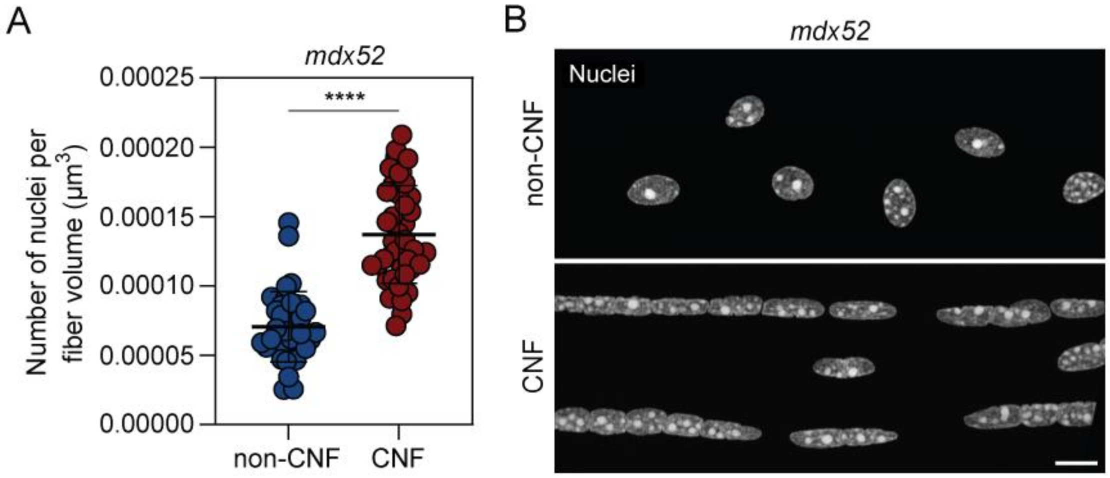

Figure S10

Nuclei numbers are increased in *mdx52* centrally nucleated myofiber segments.

(**A**) Quantification of nuclei numbers per µm^3 myofiber volume in CNF (*n*=46) versus non-CNF (*n*=41) single isolated 12-week-old *mdx52* EDL myofibers. (**B**) Representative myonuclei staining of CNF vs. non-CNF myofiber segments. Images taken at ×40 magnification, scale bar represents 10 µm. Values are mean±SD. Statistical significance was assessed by Student's *t*-test, ****P<0.0001.

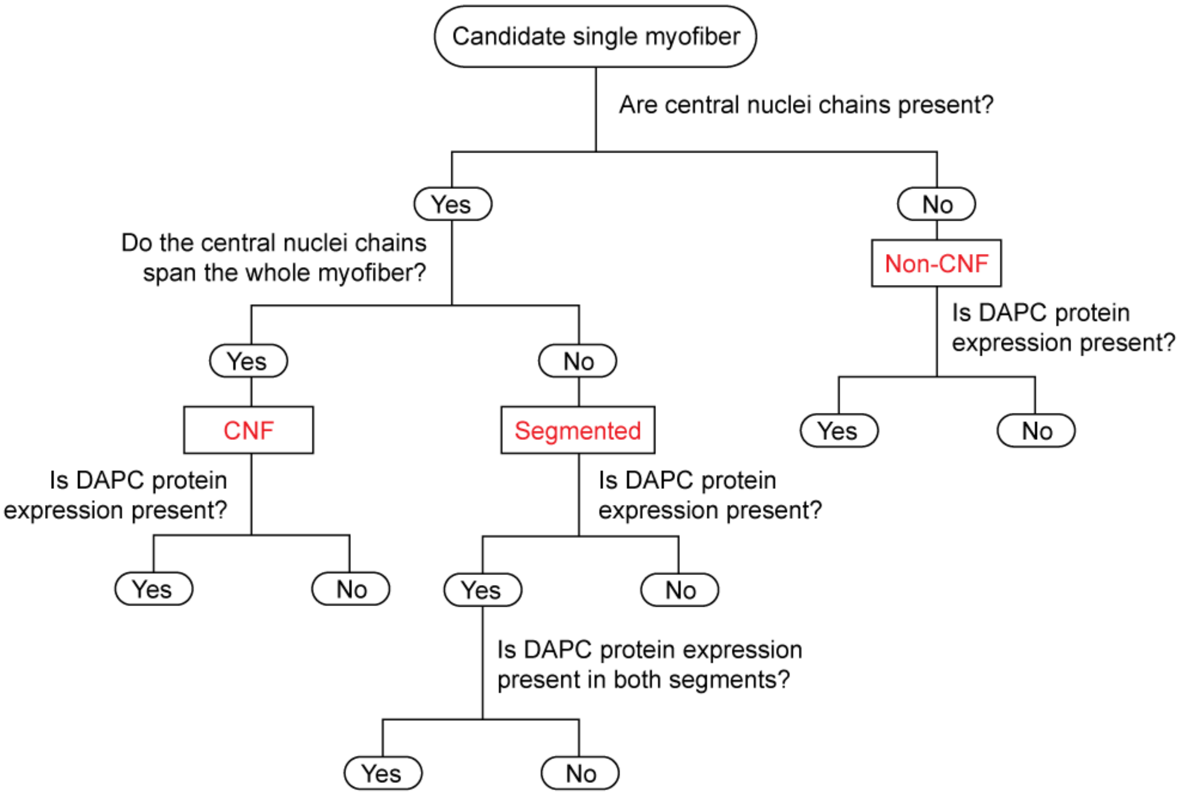

Figure S11

Schema of single myofiber classification.

Single isolated myofibers from *mdx52-Xist*$^{\Delta hs}$ mice were inspected and manually classified according to the above schema based on the degree of central nucleation and dystrophin/DAPC protein expression.

Target	Host (clone)	Product ID	Manufacturer	Dilution
**Immunofluorescence**				
**α-dystrobrevin**	rabbit pAb	α-1CTFP	In-house. Gift from Prof. K. E. Davies	1:100
**α-tubulin**	mouse mAb (AA13)	T8203	Sigma-Aldrich	1:1,000
**α-tubulin**	rat mAb (YOL1/34)	ab6161	Abcam	1:250
**α-tubulin**	rabbit mAb (EP1332Y)	ab52866	Abcam	1:250-1:500
**Dystrophin (C-terminal)**	rabbit pAb	ab15277	Abcam	1:1,000
**F-actin**	phalloidin probe conjugated with Alexa Fluor 568	A12380	Thermo Fisher Scientific	1: 200,000
**Laminin subunit α-2**	rat mAb (4H8-2)	L0663	Sigma-Aldrich	1:250
**nNOS**	rabbit mAb	ab76067	Abcam	1:100
**Telethonin**	rabbit mAb (EPR8375)	ab133646	Abcam	1:1,000
**Titin**	mouse mAb (9D10)	9 D10	DSHB	2-5 µg/ml
**Utrophin**	goat pAB	URD40	In-house. Gift from Prof. K. E. Davies	1:400-1:500
**β-Dystroglycan**	mouse mAb (43DAG1/8D5)	NCL-b-DG	Leica Biosystems	1:100
**LAMB1**	Rabbit pAb	Ab16048	Abcam	1:200
**Perlecan**	Rat mAb	A7L6	Thermo Fisher Scientific	1:1000
**Western blot**				
**Dystrophin (rod domain)**	mouse mAb (Dy4/6D3)	NCL-DYS1	Leica Biosystems	1:100
**Vinculin**	mouse mAb (hVIN-1)	V9131	Sigma-Aldrich	1:100,000
**Utrophin**	mouse mAb (8A4)	MANCHO3	DSHB	1:50

Table S1

Primary antibodies used in this study.

The anti-Utrophin antibody was obtained from the Developmental Studies Hybridoma Bank, created by the NICHD of the NIH and maintained at The University of Iowa, Department of Biology, Iowa City, IA 52242.

Secondary antibody	Product ID	Manufacturer	Dilution
**Immunofluorescence**			
**Donkey anti-goat IgG Alexa Fluor 488**	A11055	TFS	1:500-1:1,000
**Goat anti-mouse IgG Alexa Fluor-568**	A11004	TFS	1:500-1:1,000
**Goat anti-mouse IgG Alexa Fluor-647**	A21235	TFS	1:500-1:1,000
**Goat anti-mouse IgG Alexa Fluor-488**	A28175	TFS	1:500-1:1,000
**Goat anti-rabbit IgG Alexa Fluor-594**	ab150080	Abcam	1:500-1:1,000
**Goat anti-rabbit IgG Alexa Fluor-488**	A11008	TFS	1:500-1:1,000
**Goat anti-rabbit IgG Alexa Fluor-568**	A11011	TFS	1:500-1:1,000
**Goat anti-rat IgG Alexa Fluor-488**	ab150157	Abcam	1:500-1:1,000
**Goat anti-rat IgG Alexa Fluor-647**	A21247	TFS	1:500-1:1,000
**Goat anti-rat IgG Alexa Fluor-555**	A21434	TFS	1:500-1:1,000
**Western blot**			
**Horse anti-mouse IgG-HRP**	7076S	Cell Signalling	1:10,000

Table S2

Secondary antibodies used in this study.

TFS, Thermo Fisher Scientific.

Target	Product ID
mmu-miR-1a-3p	002222
mmu-miR-133a-3p	002246
mmu-miR-206-3p	000510
cel-miR-39	000200

Table S3

List of Small RNA TaqMan assays used in this study.

All assays were obtained from Thermo Fisher Scientific.

Supplementary Methods

Animal strains

Xist$^{\Delta hs}$ animals were a kind gift from Prof. Neil Brockdorff (University of Oxford).[1] *Xist*$^{\Delta hs}$ animals contain a deletion of DNase hypersensitivity region upstream of the P1 promoter of the *Xist* gene, resulting in preferential silencing of the mutation-containing chromosome. In heterozygous animals, the mutated X-chromosome is inactivated in up to 90% of the cells.[1] The *Xist*$^{\Delta hs}$ mouse has a mixed genetic background consisting of C57BL/6 and CBA.

mdx52-Xist$^{\Delta hs}$ animals were generated by crossing male *mdx52* animals with female *Xist*$^{\Delta hs}$ mice, with the resulting female F1 progeny used for experimentation.

Dystrophic *mdx52* (C57BL/6J129S-Dmd^{tm1Mok}) animals were a kind gift from Dr. Yoshitsugu Aoki (National Centre of Neurology and Psychiatry, Tokyo, Japan). The line was generated by Dr. Motoya Katsuki via targeted replacement of exon 52 in the *Dmd* gene with a neomycin resistance transgene cassette (in the antisense orientation).[2]

Wild-type C57BL/6JOlaHsd (C57BL/6) mice were obtained from Inotiv (London, England) and served as wild-type control animals. Wild-type C57BL/10J mice were used for the $BaCl_2$ injury study.

$BaCl_2$ myoinjury

Myoinjury was induced by injection of 1.2% $BaCl_2$ (Sigma-Aldrich, MO, USA) in sterile saline (total volume 20 µl) into TA muscles. Injections were performed under anaesthesia using fentanyl/fluanisone (Hypnorm, Vetapharma, Leeds, UK) and midazolam (Hypnovel, Roche, Welwyn Garden City, UK), as described previously[3]. TA muscles were macrodissected 29 days

post injury, flash frozen in liquid nitrogen-cooled isopentane, and samples stored at -80°C until ready for analysis.

Western Blot

For protein extraction, 200 TA sections (8 µm thickness) were lysed in modified Radio-Immunoprecipitation Assay (RIPA) buffer (50 mM Tris pH 8, 150 mM NaCl, 1% IGEPAL CA-630, 0.5% sodium deoxycholate, 10% SDS) containing 1× cOmplete proteinase inhibitors (Merck, NJ, USA). Samples were heated for 3 min at 100°C and centrifuged at room temperature for 10 minutes at 15,800 *g*. Protein concentration was measured using Pierce BCA Protein Assay Kit (Thermo Fisher Scientific, MA, USA) according to the manufacturer's instructions.

20-40 µg of total protein were prepared in NuPAGE LDS sample buffer supplemented with NuPAGE sample reducing agent (both Thermo Fisher Scientific) and denatured for 10 minutes at 75°C. Standards were prepared as a mix of defined different protein ratios (0-75% of wild-type dystrophin protein levels) isolated from positive control, wild-type C57 and negative control, dystrophic (*mdx52*) mouse TA. Linearity of signal was assumed for the few samples that fell outside of the standard range. All samples were loaded onto a pre-cast, NuPAGE Tris-Acetate (3-8%, Thermo Fisher Scientific) and electrophoresis run at 130 V for 1 hour 45 minutes in NuPAGE Tris-Acetate SDS Running Buffer (Thermo Fisher Scientific). Protein was electrotransferred onto 0.45 µm polyvinylidene fluoride (PVDF) membranes (Merck) for 1 hour at 30 V followed by 1 hour at 100 V in 1× NuPAGE Transfer Buffer (Thermo Fisher Scientific) supplemented with 0.1 g/l of SDS (Sigma-Aldrich) and 20% methanol. Total protein was visualized using a ChemiDoc Imaging system (Bio-Rad, CA, USA) measuring fluorescence at 700 nm. The membrane was then washed in wash solution and blocked in

blocking solution (either Odyssey blocking buffer (LI-COR Biosciences, NE, USA) or 5% milk (w/v) in tris-buffered saline buffer supplemented with 0.15 Tween-20 (v/v, TBST)). Membranes were incubated with mouse anti-dystrophin, mouse anti-utrophin or mouse anti-vinculin primary antibody (**Table S1**) overnight in blocking buffer at 4°C. Membranes were washed in tris-buffered saline buffer with 0.1% Tween-20 v/v (TBST) and incubated with anti-mouse IgG horseradish peroxidase (HRP) linked antibody (**Table S2**) in blocking buffer + 0.1% Tween-20 for 1 hour at room temperature. Chemiluminescence signal was detected using Clarity Western enhanced chemiluminescence (ECL) substrate (Bio-Rad). If membrane re-probing was necessary for the detection of proteins of similar molecular mass (e.g. dystrophin and utrophin) or using different antibodies from the same host, the membrane was stripped in 0.2 M sodium hydroxide (NaOH) for 30-120 minutes at room temperature. Subsequently, blocking step, primary and secondary antibody incubation and HRP-based detection were performed as described above.

Serum microRNA analysis

Serum miRNA analysis was performed as described previously.[4,5] Briefly, RNA was extracted from 50 µl blood serum samples using TRIzol LS (Thermo Fisher Scientific) according to manufacturer's instructions with minor modifications. A synthetic spike-in control oligonucleotide with a non-mammalian miRNA sequence (i.e. cel-miR-39, 2.5 fmol, IDT) was added at the phenolic extraction phase in order to allow for between-sample normalization. miRNAs were quantified using the small RNA TaqMan RT-qPCR method using miRNA-specific stem loop reverse transcription primers. Details of miRNA assays are listed in **Table S3**. Reverse transcription was performed using the TaqMan MicroRNA Reverse Transcription Kit (Thermo Fisher Scientific). cDNA was amplified using a StepOne Plus real-time PCR thermocycler with TaqMan Gene Expression Master Mix (both Thermo Fisher Scientific) using

universal cycling conditions: 95°C for 10 minutes, followed by 40 cycles of 95°C for 15 seconds and 60°C for 1 minute. All samples were analyzed in duplicate. Relative quantification was performed using the Pfaffl method,[6] and miRNA-of-interest abundance normalized to cel-miR-39.[5]

Microscopy

Immunofluorescence microscopy of tissue sections was performed using either wide-field Leica DMIRB Inverted Microscope with MetaMorph imaging software (Molecular Devices, CA, USA) or wide-field Leica DMi8 fluorescence microscope with LAS X Microscope Science Software Platform (all Leica Microsystems, Wetzlar, Germany). For each protein staining, optimal exposure time was chosen based on negative staining control where samples were incubated with secondary antibodies only, to account for background noise and autofluorescence of tissues. All images were processed using Fiji software.[7] Standard image processing for tissue section images included background subtraction (based on rolling ball with radius of 50 pixels), and brightness and contrast adjustment.

Single myofiber imaging was performed with ZEISS LSM 980 confocal microscope with Airyscan2 detector (ZEISS, Oberkochen, Germany). Depending on the application, the following objectives were used: 40× Plan-Apochromat oil objective (numerical aperture NA = 1.4), 25× Plan-Apochromat (NA = 0.8) or 20× Plan-Apochromat (NA = 0.8). The choice of the objective was based on the field of view and detail required in each experiment.

Image Analysis

CNF and CSA quantification in transverse tissue sections

Immunofluorescence was performed in fresh frozen TA sections using primary antibodies against α2-laminin (**Table S1**) to mark the muscle membrane, and DAPI (Thermo Fisher

Scientific) to label nuclei. The proportion of CNFs and myofiber cross-sectional area (CSA) was analysed in transverse TA muscle sections using an open-source Fiji plugin: MuscleJ2, according to developer's instructions.[8] 2-5 whole TA sections were analysed per animal. For CNF proportion analysis, values for multiple sections from the same mouse were averaged.

Classification of centrally nucleated, segmented and non-centrally nucleated myofibers

Classification into non-centrally nucleated (non-CNF), segmented, and CNFs, and assessment of DAPC protein expression was performed on myofibers isolated from *mdx52-Xist*$^{\Delta hs}$ mice (aged between 12 and 17 weeks). Myofibers were isolated and stained as described above, using the antibodies listed in **Table S1**. Each slide, was scanned using the wide-field Leica DMi8 fluorescence microscope to visualize all myofibers. Each myofiber was visually examined for nuclei (DAPI), and DAPC protein signal at the sarcolemma and classified according to a pre-defined decision schema (**Figure S11**). Briefly, myofibers were first classified into non-CNF, segmented and CNF groups based on nuclear DAPI staining. Both myofiber classes were further grouped into DAPC-expressing and non-expressing groups. Due to the observation that DAPC is present at the neuromuscular and myotendinous junctions of almost all *mdx52-Xist*$^{\Delta hs}$ myofibers, junctional sarcolemma regions were excluded from the analysis.

Analysis of the microtubule network organization

Microtubule intersection angle was analysed using TeDT direction v2017 according to the developer's instructions.[9] Two to three cortical microtubule regions per myofiber segment were analysed from z-stack images acquired at 40× magnification. Pre-analysis image processing included the z-projection of cortical microtubule region and background subtraction (radius = 50 pixels). Images were arranged so that transverse microtubules were positioned at a 90° angle

with respect to the longitudinal axis of the myofiber. The isotropic areas of microtubule nucleation surrounding myonuclei were excluded from the analysis.[9,10] In total n = 60 regions from 32 myofibers, 79 regions from 40 myofibers, and 65 regions from 31 myofibers were analysed for C57, *mdx52-Xist*$^{\Delta hs}$, and *mdx52* mice respectively. The histogram of proportion of microtubule directionality angles for each genotype was prepared using averaged and 0-1 normalized values of fractions of microtubules at 0-176° in 4° intervals.

Supplementary Results

Dystrophin expression is inversely correlated with muscle histopathology in adult *mdx52-Xist*$^{\Delta hs}$ muscles.

Histopathological analysis in adult *mdx52-Xist*$^{\Delta hs}$ TA muscles revealed the presence of abundant centrally nucleated fibers (CNFs) and foci of small diameter regenerating fibers (**Figure S2A**) at all levels of dystrophin expression, indicative of ongoing or historic muscle turnover. Mean CNF values were 11.2%, 16.7%, and 38.7% for high, medium, and low dystrophin expressing muscles, respectively (**Figure S2B**). The percentage of CNFs was strongly inversely correlated with dystrophin expression (Spearman's r=-0.86, P=0.0023, **Figure S2C**). Myofiber size distributions were similar between all analysed genotypes (**Figure S2D**).

Dystrophin expression is inversely correlated with muscle histopathology in aged *mdx52-Xist*$^{\Delta hs}$ muscles.

Histopathological analysis in aged *mdx52-Xist*$^{\Delta hs}$ TA muscles revealed abundant CNFs and foci of small diameter regenerating fibers (**Figure S3A**) at all levels of dystrophin expression. Mean CNF values were much larger than in the 6-weeks-old *mdx52-Xist*$^{\Delta hs}$ mice with 35.9%, 51.1%, and 57.1% for high, medium, and low dystrophin expressing muscles, respectively (**Figure S3B**). The percentage of CNFs was inversely correlated with dystrophin expression (Spearman's r=-0.5099, P=0.0257, **Figure S3C**), although the correlation was substantially weaker than that observed for 6-week-old animals (**Figure S3C**). Analysis of myofiber cross-sectional area revealed no differences between *mdx52-Xist*$^{\Delta hs}$ groups (**Figure S3D**). Together, these results suggest that muscles expressing dystrophin in a patchy manner continue to degenerate and regenerate throughout life, and that these pathological processes are to some extent ameliorated with higher levels of dystrophin expression.

Utrophin expression does not correlate with patchy dystrophin levels in adult and aged *mdx52-Xist*$^{\Delta hs}$ animals.

Utrophin and dystrophin exhibit reciprocal expression patterns during muscle development and dystrophic pathology.[11] Moreover, when expressed together, they bind to the same sites at the sarcolemma, as evidenced by electron microscopy detection of both proteins in muscles of transgenic mice.[12] Due to the dystrophic nature of *mdx52-Xist*$^{\Delta hs}$ myofibers, and the presence of dystrophin in positive myonuclear domains, unrestricted availability of binding sites is expected within dystrophin-negative regions. We therefore reasoned that utrophin and dystrophin might exhibit reciprocal patterns of myonuclear domain restriction.

To test this hypothesis, utrophin western blot was performed on muscle lysates from adult and aged *mdx52-Xist*$^{\Delta hs}$ animals (**Figure S4A,B**). Utrophin expression was variable but detected in all analysed animals, regardless of age or dystrophin levels. Expression of utrophin and dystrophin in TA muscles was not correlated for either 6-week-old or aged animals (Spearman's r = -0.2807, P = 0.6604, and r = -0.03506, P = 0.3023, respectively, **Figure S4C,D**).

To assess the localization of utrophin in patchy dystrophin muscles, utrophin immunofluorescence was performed in transverse TA sections from *mdx52-Xist*$^{\Delta hs}$ animals identified as exhibiting high utrophin expression within the low dystrophin expressing group (**Figure S4E**). 12-week-old *mdx52* tissue sections were used as control whereby a clear utrophin signal was detected at the membrane of relatively small, centrally nucleated myofibers organized in densely packed clusters (**Figure S4E**). Accordingly, several small groups of newly formed CNFs with positive utrophin staining were detected in aged *mdx52-Xist*$^{\Delta hs}$

muscle expressing low levels of dystrophin. Notably, no utrophin expression was observed at the sarcolemma of larger *mdx52-Xist*$^{\Delta hs}$ TA myofibers (**Figure S4E**). As such, utrophin expression in *mdx52-Xist*$^{\Delta hs}$ animals reflects the degree of ongoing muscle regeneration and is not concentrated in dystrophin-negative myonuclear domains.

Response of serum miRNA biomarkers in *mdx52-Xist*$^{\Delta hs}$ animals.

Serum microRNAs (miRNAs) have been investigated as minimally-invasive biomarkers in the context of DMD.[13] In particular, we have previously reported that serum myomiR levels are inversely correlated with dystrophin expression levels following antisense oligonucleotide-mediated exon skipping, suggesting that they may constitute promising pharmacodynamic biomarkers.[14,15] In 6-week-old animals, myomiRs were inversely correlated with dystrophin expression level (**Figure S5A-F**). Conversely, in aged animals, there was no difference between serum myomiR levels between *mdx52-Xist*$^{\Delta hs}$ animals, and accordingly no correlation with dystrophin protein expression (**Figure S5G-L**). These observations are consistent with our previous findings that serum myomiR levels are associated with regenerative pathology, which declines with age, and is effectively absent in aged animals.[16,17]

Dystrophin is expressed in post-regeneration myofibers in wild-type mice.

To assess whether muscle injury alone could induce a similar impairment in dystrophin expression, we injected adult wild-type mice (20-30-week-old, both males and females) with the muscle toxicant $BaCl_2$ in order to induce acute myonecrosis and regeneration. Animals were harvested after 29 days, after which the muscle morphology was restored but central nucleation persisted. Immunofluorescence staining in these animals revealed complete sarcolemmal dystrophin coverage, including in centrally nucleated myofibers, suggesting that

muscle regeneration *per se* does not recapitulate the phenomenon of dystrophin absence in centrally nucleated myofiber segments observed in *mdx52-Xist*$^{\Delta hs}$ mice (**Figure S8**).

Supplementary References

1. Newall AE, Duthie S, Formstone E, Nesterova T, Alexiou M, Johnston C *et al.* Primary non-random X inactivation associated with disruption of Xist promoter regulation. *Hum Mol Genet* 2001;**10**:581–589.

2. Araki E, Nakamura K, Nakao K, Kameya S, Kobayashi O, Nonaka I *et al.* Targeted disruption of exon 52 in the mouse dystrophin gene induced muscle degeneration similar to that observed in Duchenne muscular dystrophy. *Biochem Biophys Res Commun* 1997;**238**:492–497.

3. Verhaart IEC, Cappellari O, Tanganyika-de Winter CL, Plomp JJ, Nnorom S, Wells KE *et al.* Simvastatin Treatment Does Not Ameliorate Muscle Pathophysiology in a Mouse Model for Duchenne Muscular Dystrophy. *J Neuromuscul Dis***8**:845–863.

4. Roberts TC, Coenen-Stass AML, Betts CA, Wood MJA. Detection and quantification of extracellular microRNAs in murine biofluids. *Biol Proced Online* 2014;**16**:5.

5. Roberts TC, Coenen-Stass AML, Wood MJA. Assessment of RT-qPCR normalization strategies for accurate quantification of extracellular microRNAs in murine serum. *PLoS ONE* 2014;**9**:e89237.

6. Pfaffl MW. A new mathematical model for relative quantification in real-time RT-PCR. *Nucleic Acids Res* 2001;**29**:e45.

7. Schindelin J, Arganda-Carreras I, Frise E, Kaynig V, Longair M, Pietzsch T *et al.* Fiji: an open-source platform for biological-image analysis. *Nat Methods* 2012;**9**:676–682.

8. Danckaert A, Trignol A, Le Loher G, Loubens S, Staels B, Duez H *et al.* MuscleJ2: a rebuilding of MuscleJ with new features for high-content analysis of skeletal muscle immunofluorescence slides. *Skelet Muscle* 2023;**13**:14.

9. Liu W, Ralston E. A new directionality tool for assessing microtubule pattern alterations. *Cytoskeleton (Hoboken)* 2014;**71**:230–240.

10. Oddoux S, Zaal KJ, Tate V, Kenea A, Nandkeolyar SA, Reid E *et al.* Microtubules that form the stationary lattice of muscle fibers are dynamic and nucleated at Golgi elements. *J Cell Biol* 2013;**203**:205–213.

11. Mizuno Y, Nonaka I, Hirai S, Ozawa E. Reciprocal expression of dystrophin and utrophin in muscles of Duchenne muscular dystrophy patients, female DMD-carriers and control subjects. *J Neurol Sci* 1993;**119**:43–52.

12. Culle MJ, Walsh JM, Tinsle JM, Fisher R, Davies KE. Immunogold confirmation that utrophin is localized to the normal position of dystrophin in dystrophin-negative transgenic mouse muscle. *Histochem J* 2001;**33**:579–583.

13. Coenen-Stass AML, Wood MJA, Roberts TC. Biomarker Potential of Extracellular miRNAs in Duchenne Muscular Dystrophy. *Trends Mol Med* 2017;**23**:989–1001.

14. Chwalenia K, Oieni J, Zemła J, Lekka M, Ahlskog N, Coenen-Stass AML *et al.* Exon skipping induces uniform dystrophin rescue with dose-dependent restoration of serum

miRNA biomarkers and muscle biophysical properties. *Molecular Therapy - Nucleic Acids* 2022;**29**:955–968.

15. Roberts TC, Godfrey C, McClorey G, Vader P, Briggs D, Gardiner C *et al.* Extracellular microRNAs are dynamic non-vesicular biomarkers of muscle turnover. *Nucl Acids Res* 2013;**41**:9500–9513.

16. Coenen-Stass AML, Betts CA, Lee YF, Mäger I, Turunen MP, El Andaloussi S *et al.* Selective release of muscle-specific, extracellular microRNAs during myogenic differentiation. *Hum Mol Genet* 2016;**25**:3960–3974.

17. Coenen-Stass AML, Sork H, Gatto S, Godfrey C, Bhomra A, Krjutškov K *et al.* Comprehensive RNA-Sequencing Analysis in Serum and Muscle Reveals Novel Small RNA Signatures with Biomarker Potential for DMD. *Mol Ther Nucleic Acids* 2018;**13**:1–15.

18. Denes LT, Kelley CP, Wang ET. Microtubule-based transport is essential to distribute RNA and nascent protein in skeletal muscle. *Nat Commun* 2021;**12**:6079.

19. Scarborough EA, Uchida K, Vogel M, Erlitzki N, Iyer M, Phyo SA *et al.* Microtubules orchestrate local translation to enable cardiac growth. *Nat Commun* 2021;**12**:1547.

20. Prins KW, Humston JL, Mehta A, Tate V, Ralston E, Ervasti JM. Dystrophin is a microtubule-associated protein. *J Cell Biol* 2009;**186**:363–369.

21. Belanto JJ, Mader TL, Eckhoff MD, Strandjord DM, Banks GB, Gardner MK *et al.* Microtubule binding distinguishes dystrophin from utrophin. *Proc Natl Acad Sci U S A* 2014;**111**:5723–5728.

22. Percival JM, Gregorevic P, Odom GL, Banks GB, Chamberlain JS, Froehner SC. rAAV6-microdystrophin rescues aberrant Golgi complex organization in mdx skeletal muscles. *Traffic* 2007;**8**:1424–1439.

23. Collins BC, Shapiro JB, Scheib MM, Musci RV, Verma M, Kardon G. Three-dimensional imaging studies in mice identify cellular dynamics of skeletal muscle regeneration. *Dev Cell* 2024;**59**:1457-1474.e5.

24. Meyer GA. Evidence of induced muscle regeneration persists for years in the mouse. *Muscle Nerve* 2018;**58**:858–862.

25. Guiraud S, Edwards B, Squire SE, Moir L, Berg A, Babbs A *et al.* Embryonic myosin is a regeneration marker to monitor utrophin-based therapies for DMD. *Hum Mol Genet* 2019;**28**:307–319.

26. Massopust RT, Lee YI, Pritchard AL, Nguyen V-KM, McCreedy DA, Thompson WJ. Lifetime analysis of mdx skeletal muscle reveals a progressive pathology that leads to myofiber loss. *Sci Rep* 2020;**10**:17248.

27. Duddy W, Duguez S, Johnston H, Cohen TV, Phadke A, Gordish-Dressman H *et al.* Muscular dystrophy in the mdx mouse is a severe myopathy compounded by hypotrophy, hypertrophy and hyperplasia. *Skeletal Muscle* 2015;**5**:16.

28. Echigoya Y, Lee J, Rodrigues M, Nagata T, Tanihata J, Nozohourmehrabad A *et al.* Mutation types and aging differently affect revertant fiber expansion in dystrophic mdx and mdx52 mice. *PLoS One* 2013;**8**:e69194.

29. Bugnard E, Zaal KJM, Ralston E. Reorganization of microtubule nucleation during muscle differentiation. *Cell Motil Cytoskeleton* 2005;**60**:1–13.
